# Post-occupancy evaluation of former residences of celebrities in Yangzhou based on the comparison of online reviews and questionnaire data

**DOI:** 10.1371/journal.pone.0357793

**Published:** 2026-09-03

**Authors:** Kexin Wei, Ziyang Wang, Xinyu Duan, Wei Liu, Yueling Xiao, Rong Zhu

**Affiliations:** School of Design, Jiangnan University, Wuxi, Jiangsu, China; Macau University of Science and Technology, MACAO

## Abstract

Online review data and traditional questionnaire data are common data sources in the current field of cultural heritage research. The former features a large data volume yet suffers from obvious limitations such as incompleteness, algorithm interference, dirty data and sensitivity; the latter enjoys a high degree of quantification but is constrained by high costs and limited sample size. To explore the complementary mechanism of the two types of data, this study selects 9 national-level former residences of celebrities in Yangzhou as cases and adopts a sequential mixed-methods design. First, content analysis and thematic coding are conducted on online reviews to identify 5 major categories of negative evaluations, based on the coding results, an indicator system is constructed, and offline questionnaire data are collected for AHP-FCE quantitative analysis. The results show that the two types of data exhibit partial correspondence in diagnosing core shortcomings, which, to a certain extent, reflects the inherent logic of the sequential design in which the questionnaire indicators were derived from the online review coding. Cultural expression, interactive experience, accessibility, and signage facilities are jointly identified as the weakest dimensions. Online data provide detailed descriptions and situational characteristics, while questionnaire data reflect the weight and severity of existing problems. Meanwhile, discrepancies exist between the two datasets. Complaints about service attitude appear frequently in online reviews, while this item scores well in questionnaires, reflecting the difference between extreme individual cases and overall public perception. Overall, online review data can truly and centrally reflect systematic defects, possessing unique advantages in capturing specific details and users’ real emotional perceptions. Questionnaire survey data can reflect the severity of problems with strong pertinence through weight analysis. The integrated management and diagnosis framework combining the two approaches provides timely and insightful decision-making support for the targeted optimization of cultural heritage sites, in which the granularity differences between the two data types offer a unique perspective for understanding tourist experiences.

## Introduction

Former residences of celebrities are places where socially influential historical figures once lived, in which physical forms and cultural significance of their lives, work, and social interactions are preserved [1]. These residences form part of social cultural memory [2]. With social changes, former residences of celebrities have gradually shifted from commemorative sites to complexes bearing functions such as cultural dissemination, educational display, and tourism development, have become important cultural tourism destinations [3], and promoted urban cultural tourism construction and regional economic development [4].

Maintaining the holistic environment and architectural form of former residences of celebrities, utilizing digital technology to provide virtual experiences, and recreating cultural memory to stimulate intellectual and emotional resonance of tourists are considered key focuses of utilisation of cultural heritage [5,6]. The preservation of physical form and cultural significance helps improve tourism development level of former residences of celebrities [7], but the deep social connections between them as cultural transmission carriers and the public have not been fully considered. Utilisation of cultural heritage is a process of weighing historical culture against real-world development [8]. Regardless of the development purpose, “use” is the essence. Public experience is one of the metrics for judging development outcomes [9], and provides possible references for utilisation of cultural heritage [10].Post-Occupancy Evaluation (POE) refers to the process of systematically assessing built environments that have been put into use, with a focus on user satisfaction, functionality, as well as users’ physical and mental health [11]. In the context of cultural heritage tourism, POE provides a structured framework for assessing the extent to which heritage sites can meet tourists’ needs and expectations beyond physical conservation [12]. As representatives of local cultural heritage, former residences of celebrities are not static spaces, but cultures continuously shaped by public participation [13]. However, public usage feedback and actual needs regarding former residences of celebrities have not been sufficiently studied.

Traditionally, research on public feedback regarding cultural heritage site s relied on field surveys [14,15]. For example, Likert scales are widely used in public interviews and questionnaire surveys to measure public satisfaction with tourist destination development [16]. With the popularity of online travel platforms, the public has become accustomed to sharing comments and exchanging views online after visiting to cultural heritage sites [17]. These forms of subjective perception of cultural heritage sites include various elements such as images, text, and videos, which reflect the tourism experience, satisfaction, and behavioral tendencies of the public [18]. They are characterized by fast updates, high shareability, and good accessibility [19], thus becoming an important reference for public travel decisions [20] and providing richer data samples for researchers [21,22].

Text analysis was the first method used for analyzing review data on travel websites [23]. This method was implemented with the help of tools such as Python and ROST CM6.0, which can extract high-frequency words from the text information of online reviews, conduct word frequency statistics, and analyze high-frequency word co-occurrence networks, thereby quickly locating public focus points and revealing the internal connections between evaluation elements [24]. Liu and Guo compared high-frequency words of text information from online travel platforms to reveal changes in the image perception of ice and snow tourism destinations in China following the Beijing Winter Olympics [25]. Subsequently, sentiment analysis based on the emotional intensity of text output emerged. Based on sentiment dictionaries, the emotional preference of reviews was calculated to reveal implied positive or negative emotions, thereby quantifying public satisfaction [26]. Guerrero-Rodriguez et al. analyzed online travel reviews to determine the emotional perception of cultural destinations in Mexico [27]. Structured quantitative analysis through content coding and category induction can further determine the composition of specific emotional tendencies, thus deconstructing the categories of emotional attribution. The Grounded Theory can conceptualize text, deeply analyze the structural relationships between various emotional evaluation elements, and extract elements that represent emotional evaluations of tourists on attractions, and enables construction of a three-level bottom-up coding process to derive core comment categories, thus forming a theoretical framework explaining the emotional characteristics in tourist reviews [28,29]. For example, Jianqiang Yin coded online review data of Macau tourist destinations based on the Grounded Theory to extract the perception of tourists for Macau image and the potential influencing mechanisms of positive and negative emotions [30]. With the development of AI technology, it is possible to process large-scale datasets and rapidly analyze text information. Some research has begun to employ machine learning models [31] and deep learning models [32] for thematic clustering analysis of language models quantitatively and structurally, to identify the categories and emotional characteristics of public evaluations. Although AI technology can quickly analyze huge amounts of data information, the results need to be manually reviewed [33].

Travel website reviews influence the public’s travel decisions and perceptions for tourist destinations [34]. Positive reviews can enhance public satisfaction, dependency, and loyalty towards tourist destinations [35]. Negative or distorted reviews can increase perceived risk of the public, affect their willingness to visit, and shape a negative image, thereby hindering the realization of potential utilization or optimal economic development of tourist attractions [36]. Therefore, special attention shall be paid to negative reviews to mitigate the risk of the public making unfavorable decisions [37,38].

Different research methods capture different facets of social reality [13]. Overall, as two primary research approaches for public participation in tourist destinations, both online review data analysis and traditional questionnaire surveys have their inherent limitations. Online review data are recognized for their advantages of massive volume, continuity and non-reactivity. Although existing studies have preliminarily verified the value of online review data in tourism satisfaction assessment, their inherent characteristics such as incompleteness, algorithm interference, dirty data, sensitivity and restricted accessibility remain key concerns that need to be addressed [39–41]. For instance, relevant research has confirmed that the quality of online review data varies greatly, and false information may exist [42]. Such data often only reflect the experiences of partial users, leading to data unfairness [43]. Meanwhile, they are widely questioned for failing to meet the basic principles of repeatability and reliability [44]. In contrast, traditional questionnaire surveys entail high time and economic costs. The sample size collected in research is usually limited, making it difficult to conduct large-scale research [45]. To overcome the epistemological limitations of a single research method and reflect the authenticity and diversity of public participation, this study proposes the following core research questions:(1) How can the respective strengths of the two types of data be utilized to compensate for each other’s shortcomings? (2) Can the integration of the two datasets generate new insights beyond what a single method can achieve?Accordingly, this study adopts a sequential mixed-methods design. Taking 9 national-level former residences of celebrities in Yangzhou as cases, it collects online reviews and offline questionnaire data. By employing content analysis, thematic analysis, and the AHP‑FCE method, this paper systematically compares the similarities and differences between the two data sources across evaluation dimensions. The research findings not only provide a basis for the targeted improvement of former residences of celebrities in Yangzhou, but also offer a dual‑track complementary mixed analysis paradigm combining online data and traditional surveys for the revitalization and management of cultural heritage in similar historic cities.

## Methodology

### Objects

Yangzhou boasts not only a wealth of cultural heritage sites but also a substantial tourist catchment area. In 2024, Yangzhou received a total of 119 million domestic tourists, a year-on-year increase of 20.8%, with the growth rate ranking first in Jiangsu Province, driving the annual total tourism revenue up to RMB 103.688 billion [46]. The *2025 Qingming Festival Tourism Forecast Insight* showed Yangzhou ranked among the top ten cities on the national list of popular tourist destinations [47]. This indicates that the attractiveness of tourist attractions in Yangzhou to tourists continues to rise. As a famous Chinese historical and cultural city, Yangzhou was an important national commercial and trade center and transportation hub historically, attracting many celebrities to reside here and possessing many former residences of celebrities. As of October 2024, there were 117 former residences of celebrities, with 10 having been designated as national-level cultural relics preservation units. The Yangzhou Municipal People’s Government launched an all-for-one tourism scheme since May 2021, with various tourist attractions fully open to tourists. Many former residences of celebrities have been renovated and reopened to the public.

**Fig 1:**
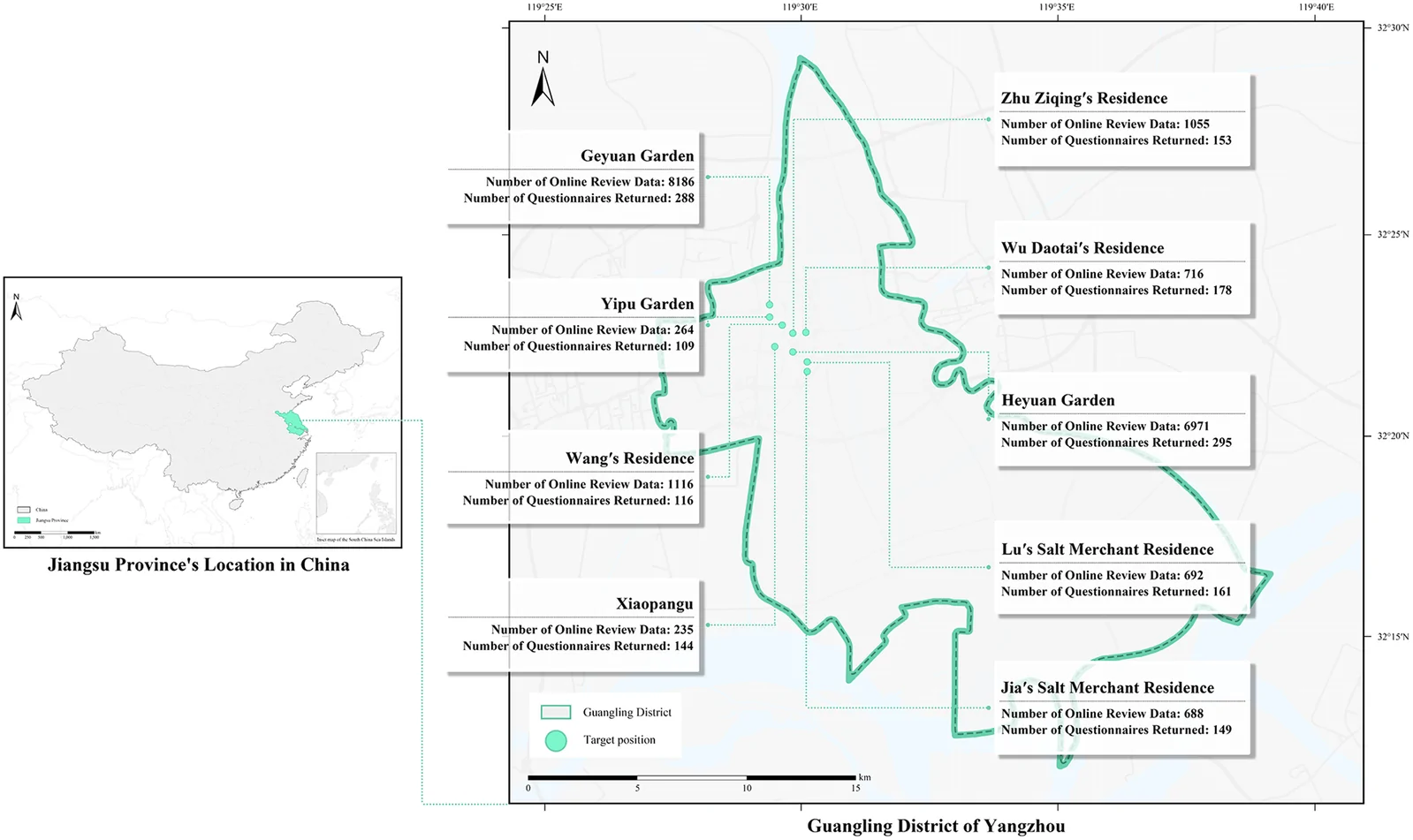
Scope of the study and amount of data (Base map from natural Earth data (public domain, https://www.naturalearthdata.com/)).

To ensure sufficient data volume and representativeness, 9 of these national-level residences were selected as cases in this study. The selection of research subjects not only considered tourism popularity and data availability but also encompassed different planning functional zones to ensure the representativeness of the data for cultural heritage site planning decisions. Among them, Heyuan Garden, Geyuan Garden, and Wang’s Residence, as national 4A-level attractions, can receive over 250,000 tourists monthly per site. Other selected residences are also popular tourist attractions in Yangzhou. For example, Zhu Ziqing’s Residence received 24,833 tourists during the 7-day Spring Festival period in 2024. It is evident that the sustained attraction and potential to generate considerable online review data can provide the core data foundation for this study. It is worth noting that the remaining national-level former residence, the Wang’s Salt Merchant Residence, was not included in this study due to a relatively short period of being open to the public after restoration and the consequently insufficient amount of data samples. In summary, these 9 national-level former residences of celebrities, with significant tourism popularity and abundant online review data, provide an ample sample basis for this study (Fig 1).

### Data source

Online data was mainly collected from mainstream Chinese travel and consumption websites, including Mafengwo, Qunar, Ctrip, and Dianping. Ctrip, founded in 1999, has now become the largest travel website in China [20]. In March 2023, Chinese active users of Ctrip reached 83.941 million. Qunar, established in February 2005, is the world’s largest online Chinese travel website. In March 2023, active users of Qunar reached 27.036 million. Mafengwo, founded in 2006, is a travel content sharing platform featuring “content + transaction,” with an average monthly active users up to 8.7078 million. Dianping is the first Web 2.0-based local search portal in China, launched in April 2003. As a well-known consumption website in China, it boasts a massive user base and consumer-generated content reviews, including reviews of tourist attractions, travel guides, and travel photos [27]. In May 2025, active users of Dianping reached 213.4005 million. The aforementioned websites have a wide user base and the fundamental potential to provide rich evaluation data sources. To ensure the research authenticity and comprehensiveness, review data from the above 4 website platforms regarding the 9 representative former residences of celebrities in Yangzhou between May 1, 2021 (the start of all-for-one tourism policy in Yangzhou) and October 1, 2025 were collected for this study.

The online review data were collected manually from publicly accessible pages of the four platforms. No automated tools were employed. The collection complied with each platform’s terms of service and robots.txt, and only non‑personal, publicly available information was extracted. The authors believe this method does not violate any applicable terms or conditions.

Offline data was collected by on-site questionnaires. From December 30, 2025 to February 25, 2026, tourists were intercepted at the exits of the 9 former residences of celebrities using a systematic random sampling method. The specific sampling procedure was as follows: 1 tourist was selected at an interval of every 5 departing visitors and invited to complete a paper questionnaire on site. If the selected tourist refused to participate or terminated the interview halfway, the next tourist was selected in sequence. A minimum of 50 valid questionnaires per survey day at each former residence was set as the sampling target, with a total of 4 rounds conducted (each round covering all sites). In actual implementation, due to variations in tourist flow across different former residences during certain rounds, some sites did not fully achieve the target value. Consequently, the total number of questionnaires retrieved was 1,724 (Table 1). After excluding incomplete responses, logically contradictory entries, and questionnaires completed in an unreasonably short time, 1,643 valid samples were retained, yielding a valid questionnaire rate of 95.3%.

**Table 1:** Questionnaire collection numbers by round and site.

Site	Round 1	Round 2	Round 3	Round 4	Subtotal (valid)
Heyuan Garden	55	52	55	51	213 (203)
Geyuan Garden	52	55	51	52	210 (199)
Wang’s Residence	49	50	48	51	198 (190)
Yipu Garden	47	47	43	46	183 (174)
Jia’s Salt Merchant Residence	45	44	44	45	178 (170)
Lu’s Salt Merchant Residence	47	46	46	47	186 (176)
Zhu Ziqing’s Residence	48	49	47	49	193 (185)
Wu Daotai’s Residence	49	48	47	49	193 (185)
Xiaopangu	46	42	40	42	170 (161)
**Total (by round)**	**438**	**433**	**421**	**432**	**1,724 (1,643)**

### Methodology and procedures

**Fig 2:**
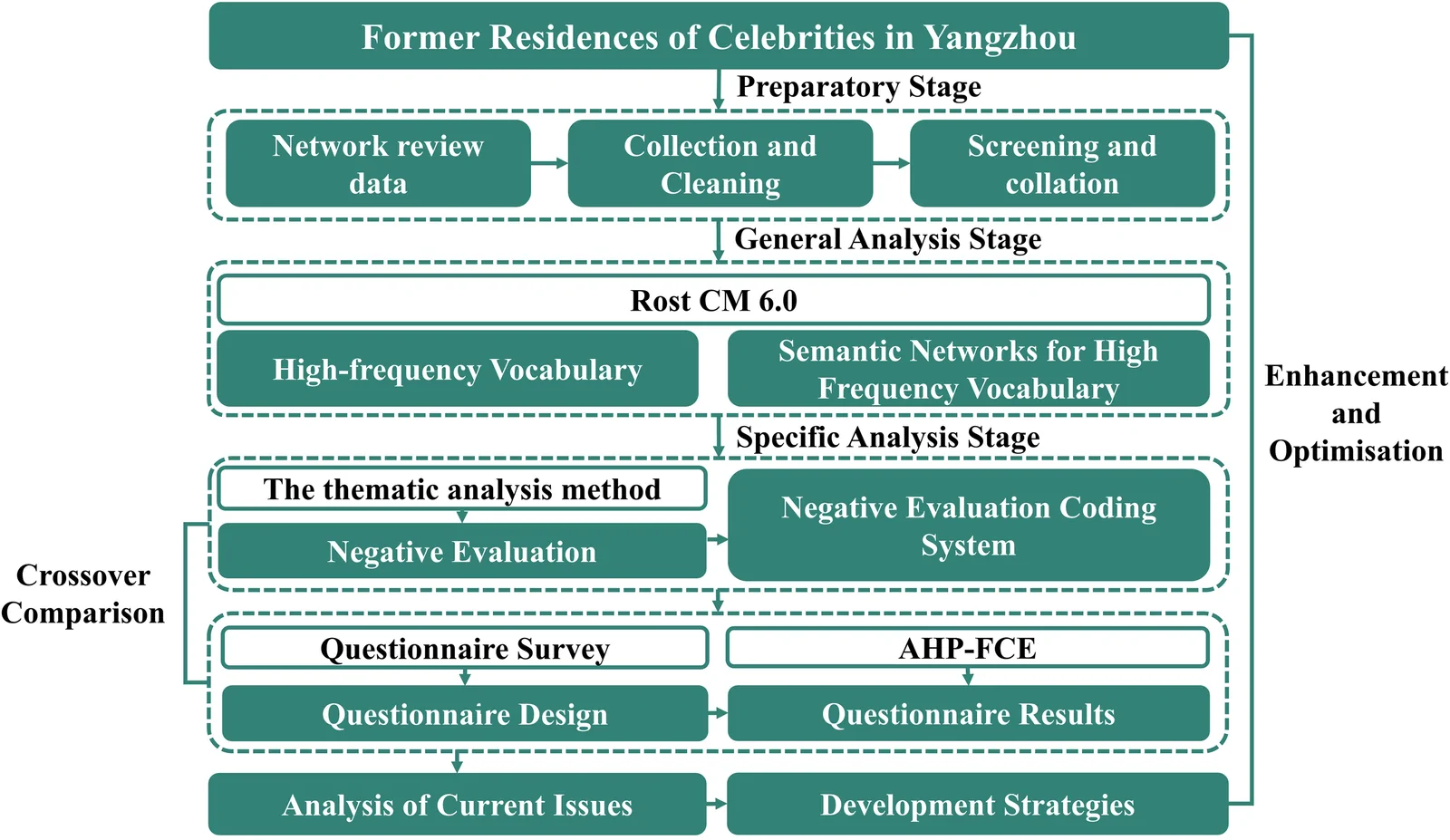
Research process.

This study adopts a sequential mixed-methods design [48], which mainly consists of two stages. In the first stage, content analysis and thematic coding of online review data are conducted to tentatively identify the core negative dimensions and specific manifestations of post-occupancy evaluation for former residences of celebrities in Yangzhou. The coding results form a preliminary analysis of existing problems. In the second stage, a structured questionnaire is designed based on the analytical results. Offline surveys are then carried out to quantitatively assess the prevalence, severity and relative weight of these problems. By comparing the two types of data, the research attempts to integrate their respective strengths, thereby forming a more comprehensive diagnosis than that relying on a single data source (Fig 2).

This study was approved by the Medical Ethics Committee of Jiangnan University (Approval No. JNU202509RB046). Oral informed consent was obtained from all participants prior to the questionnaire survey, and the requirement for written informed consent was waived by the ethics committee.

#### Stage I: Analysis of online review data.

The research adopted the content analysis method. The content analysis method was a research approach that analyzes documentary content through objective, systematic and quantitative description. The specific procedures were as follows:

(1) Online review data was collected manually. Two trained researchers browsed and extracted reviews one by one from the publicly accessible pages of Mafengwo, Qunar, Ctrip, and Dianping between November 2025 and December 2025.The collection process was strictly limited to publicly accessible review texts and publication dates that could be viewed without user login. The researchers only copied the review text content and did not collect users’ nicknames, registered IDs, avatars, or any personal profile information. To ensure data quality and privacy compliance, manual desensitisation was performed simultaneously during the collection stage. If any personal identifiable information such as phone numbers, email addresses, or ID numbers was found in the review text, it was immediately replaced with a “[desensitised]” mark. All extracted reviews were uniformly assigned anonymous codes (C-001 to C-n) when entering the analysis database, avoiding any association with the original publishers. At the same time, invalid review data such as duplicates, English characters, and emoticon characters was removed. After cross-checking and deduplication, a total of 19,923 valid review samples were obtained.
(2) Collected text information was analyzed using content analysis method, with the aid of ROST CM6.0 software. ROST CM 6.0 is a text mining software developed by Wuhan University, China [49], designed for organizing, indexing, retrieving, and applying online text data, characterized by scalability, intelligence, and objectivity [50]. The analysis covered high-frequency words, high-frequency word semantic networks, and sentiment tendency.
Data underwent word segmentation processing. In particular, Chinese text was accurately segmented with built-in and custom dictionaries, and stop words such as “的” (de), “了” (le), “和” (he) were removed. Through the word frequency statistics, high-frequency word was analyzed, and core elements related to the experience of former residences of celebrities were identified. By leveraging the “social network and semantic network analysis” function of the software, a co-occurrence matrix of high-frequency words was constructed based on co-occurrence (the frequency of words appearing together within the same review or a set window). By analyzing metrics such as density, centrality, and cohesive subgroups, the inherent relations between different themes and elements in tourist reviews were identified.
(3) Combined with word frequency analysis and semantic network analysis, a hybrid strategy of “manual identification as the primary method and automatic screening as the auxiliary method” was adopted to extract negative reviews, forming a clear material pool for subsequent thematic analysis. First, two researchers with a research background in cultural heritage tourism independently read all online reviews. They judged the emotional tendency of each review according to its semantic content, and marked those explicitly expressing dissatisfaction, criticism or negative emotions. Meanwhile, the sentiment analysis module of ROST CM6.0 software was used to calculate the distribution of positive, neutral and negative emotions in the reviews. However, its built-in general sentiment dictionary lacks contextual adaptability tailored to cultural heritage scenic sites, which easily leads to misjudgment of textual sentiment. Conversational online reviews such as short sentences, rhetorical questions and contextual irony are also highly prone to misclassification by machine sentiment scoring. To ensure the accuracy of negative sample screening, the software screening results were only used as auxiliary reference. The manually labeled results were compared with the automatically screened candidate set. Reviews with consistent results from both methods were included in the negative review dataset. Inconsistent entries were judged by a third researcher. Finally, a total of 3,922 negative reviews were obtained, accounting for 19.7% of all reviews. This mixed screening method balances efficiency and accuracy. The manual review process can effectively correct errors in automatic analysis and identify complex emotional expressions in the text.
(4) The thematic analysis method was adopted [51–54]. Two researchers with academic backgrounds in cultural heritage tourism were selected as coders to code the screened collection of negative reviews. On this basis, a negative evaluation system for former residences of celebrities in Yangzhou was established, so as to interpret the deficiencies in the current conservation and tourism development of these historic sites. The specific procedures are as follows:

Initial coding. The two coders independently read all negative reviews and adopted a “open labeling” approach to extract initial concepts that reflect negative evaluations. For example, the original comment “The whole garden can be toured in 15 min and there is nothing much to see” was coded as “excessively short visiting duration” (Table 2). The coding unit in this study was the semantic segment within each review. When a single review contained multiple independent statements, it could be divided into multiple semantic units, each of which was coded separately. Therefore, a single review could contribute multiple coded mentions under different themes. To avoid ambiguity at the coding-unit level, each semantic unit was assigned only to one most appropriate initial code, sub-theme, and core theme; this does not mean that an entire review was limited to only one coded mention.
Theme identification. Similar initial concepts were merged to form sub-themes. For instance, concepts including “excessively short visiting duration”, “limited accessible area”, and “scattered and insufficient exhibits” were categorized under the “monotonous exhibition content”. Sub-themes were further integrated to identify higher-level core themes. For example, “monotonous exhibition content”, “building damage”, and “poor preservation of indoor cultural relics” were grouped into the “scenic area opening and exhibition design” (Table 2).
Theme review. A comprehensive review was conducted on the preliminarily aggregated sub-themes and core themes. The coding attribution was verified one by one against the original review dataset. Redundant themes inconsistent with textual facts were eliminated, and mismatched or confused coding entries were revised.
Theme definition and naming. Combined with the research context and the core semantic meaning of the texts, the specific connotation of each theme was clearly defined. All retained themes were given standardized definitions and formal names. Finally, a well-structured hierarchical framework of negative evaluation themes was established.

**Table 2:** Coding examples.

Original comment (Chinese)	Initial coding	Sub-theme	Core theme
“The whole garden can be toured in 15 min and there is nothing much to see”	Excessively short visiting duration	Monotonous exhibition content	Accessibility and exhibition design
“There are no signboards at the entrance, and it cannot be located via navigation apps”	Poor accessibility and inaccurate navigation	Insufficient transport and surrounding supporting facilities	Facility development
“The ticket seller has a very bad attitude and is indifferent to visitors”	Rude attitude of ticket staff	Poor staff attitude and skills	Management, operation and maintenance
“The admission ticket costs RMB 40, yet there are only a few empty rooms inside”	Overpriced ticket and low cost performance	Confused consumption price	Management, operation and maintenance
“Claimed as a former salt merchant’s residence, but it is largely occupied by Huaiyang restaurants”	Excessive commercialization	Unclear distinguishing features of attractions	Tourist experience and activity engagement

In addition, this study examined inter-coder reliability at each hierarchical level to ensure analytical objectivity. The percentage agreement of initial coding was 86.2%, with a Cohen’s Kappa coefficient of 0.82; the consistency of sub-theme coding rose to 91.0%, with a Kappa coefficient of 0.86; the consistency of core theme categorization reached 95.7%, with a Kappa coefficient of 0.91, indicating a good level of inter-coder agreement.

A standardized calibration procedure was established to resolve coding discrepancies. First, the two coders conducted cross-review and semantic discussion on all questionable entries. When the two coders failed to reach an agreement, a third researcher with a research background in cultural heritage tourism was consulted to make a final judgment by combining the textual context and research definitions. The number of disputed coding entries, the causes of disagreements, and the calibration basis were fully recorded throughout the process.

The four online platforms differ significantly in user composition and community culture. Although this study adopted open-ended free-text reviews instead of structured indicators preset by the platforms, a stratified heterogeneity test across platforms was conducted to examine whether platform differences would affect the core research findings. All data platforms were classified into two categories: local life-oriented platforms (Dianping) and long-distance travel-oriented platforms (Mafengwo, Ctrip and Qunar). Independent coding and thematic statistical comparison were conducted for each subset of different platforms respectively. The results reveal that the core thematic structure of negative reviews across the four major platforms is highly convergent. Key shortcomings such as exhibition content, landscape environment, service management and supporting facilities are widely reflected on all platforms, with only a few idiosyncratic differences existing in secondary dimensions including price perception and travel services. Overall, structural differences across platforms exert limited influence on the core negative evaluation system, indicating that the aggregated full sample possesses sound representativeness.

#### Stage II: Analysis of offline questionnaire data.

Questionnaire surveys were conducted to collect offline tourists’ post-occupancy evaluation data on the former residences of celebrities in Yangzhou, and the AHP-FCE method was adopted for result analysis. This method can hierarchically decompose elements relevant to the research objectives, and conduct comprehensive evaluation calculation through pairwise comparison and fuzzy mathematics [55]. It is suitable for handling the multi-level evaluation indicators and uncertain information in the post-occupancy evaluation of former residences of celebrities in Yangzhou, yielding systematic and intuitive evaluation results (The detailed calculation process is shown in S1 File).

(1) Construction of evaluation indicators (see Table S1-1 in S1 File). To explore the complementary mechanism of the two types of data, evaluation indicators were formulated based on the negative factor elements extracted from online reviews. The overall target layer was set as “Post-Occupancy Evaluation of Former Residences of Celebrities in Yangzhou”, with criterion-layer indicators and sub-criterion-layer indicators established correspondingly. The full questionnaire is provided in S2 File.
(2) Construction of evaluation matrix and determination of weights (see Table S1- 2 in S1 File). 15 experts with diverse professional backgrounds were invited to conduct pairwise comparisons and assign weights to indicators at all levels. The selection criteria for experts were as follows: (a) Having at least five years of working experience in the fields of heritage tourism, landscape architecture or cultural policy; (b) Having direct practical participation or academic research experience related to former residences of celebrities in Yangzhou; (c) Being willing to participate in the evaluation process. The composition of the expert panel is as follows: 5 landscape designers (including 2 members of local design research institutes and 3 designers from private design companies), 6 academic researchers from universities (all professors or doctoral researchers specializing in cultural heritage conservation or cultural tourism development), and 4 representatives from local government cultural and tourism authorities as well as members of local folk cultural research organizations (including 2 staff from Yangzhou Municipal Bureau of Culture, Radio, Television and Tourism, and 2 members or consultants of local folk cultural heritage conservation organizations). In terms of gender distribution, there are 9 female experts and 6 male experts. All experts are familiar with at least 2 of the 9 former residences involved in this study.
Inter-expert consistency was assessed by comparing the geometric mean of all judgment matrices with the deviations of individual matrices [56]. The consistency ratio (CR) between each expert’s judgment matrix and the aggregated matrix was calculated. If an expert’s CR value was > 0.1, the internal logic of their judgment matrix was regarded as inconsistent and thus eliminated. The standard deviation of expert scores at each comparison position was calculated. Where the standard deviation of a certain position was > 2 (an excessive discrepancy in scale values), relevant experts were consulted for confirmation or invited to rescore [57]. In this study, the standard deviation of all comparison positions was ≤ 1.5, suggesting that inter-expert discrepancies remained within an acceptable range. The calculation results show that the consistency ratios (*CR*) of the judgment matrices of all 15 experts were below the threshold of 0.1, with the highest *CR* being 0.067. Therefore, no expert matrix was excluded. The final weights were determined using the geometric mean of the responses from all 15 experts, which effectively mitigated the influence of individual extreme values. Combined with the 1–7 scale method, indicators at all levels were compared and assigned scores layer by layer to construct judgment matrices (see Table S1-4 in S1 File). Weight values were determined through calculation, and consistency tests were performed on all judgment matrices. Meanwhile, to test the robustness of the weights, this study conducted a sensitivity analysis based on expert grouping. 15 experts were divided into three groups based on professional background: landscape designers, scientific researchers, and local administrators. Judgment matrices were independently constructed, indicator weights and comprehensive evaluation values were calculated for each group, and differences in weight distribution among different expert groups were compared. The comparison of multiple groups of results reveals that the weights obtained by experts with different professional standpoints show an overall consistent trend, and no changes occur in the evaluation grade and ranking of samples. It indicates that the weight results of this study have low dependence on the composition of the expert panel, and the evaluation conclusions are stable and reliable.
(3) A Likert scale was adopted to establish the evaluation set (V={Excellent, Good, Average, Poor, Very Poor}={100, 80, 60, 40, 20}). This equal-value assignment method has been widely applied in existing studies on cultural heritage and tourism satisfaction [55,58]. The advantage of equidistant value assignment lies in facilitating the calculation of weighted average scores [59,60] and enabling straightforward interpretation of results. Finally, evaluation data were collected by distributing questionnaire surveys to tourist groups. The membership degrees of each evaluation factor corresponding to the evaluation set were calculated, and the grade fuzzy membership degrees as well as the fuzzy membership matrix were constructed accordingly. Fuzzy comprehensive evaluation scores were obtained through multi-level fuzzy comprehensive operation (For the FCE calculation process and final results, see Table S1-6 in S1 File). The score ranking can intuitively and accurately reflect the performance of each evaluation indicator, which allows for comparison with online review results and provides a reference for subsequent design optimization.
(4) Based on the above analysis, this paper identifies the deficiencies in the current utilization of former residences of celebrities in Yangzhou and puts forward corresponding attraction design strategies.

### Analysis of online review results regarding former residences of celebrities in Yangzhou

#### High-frequency word analysis.

Word frequency of collected text was extracted with ROST CM6.0 software. After meaningless and ambiguous words being removed, and words with similar meanings being merged, 200 high-frequency words were ultimately extracted for each tourist attraction. Limited by article length, only the top 20 high-frequency words for each attraction are listed (For details, please see S1 Table in the supplementary materials). High-frequency words represent the impression of the tourist attraction for most tourists. The higher the frequency, the deeper the impression.

Based on the high-frequency word analysis of online reviews, it was evident that the post-occupancy evaluation regarding former residences of celebrities in Yangzhou consists of scenic elements, tourism services, transportation methods, cultural themes, exhibition arrangements, management services, seasonal climate, etc. Negative reviews mainly focused on the following aspects:

First, vocabulary related to attraction scale: Words such as “Area” repeatedly appeared in reviews of various former residences, and often co-occurred with experiential descriptions including “Be affected by” “Not often”.
Second, vocabulary related to accessibility and tourism services: Terms like “Lane”, “Locality”, “Free”, “Convenient”, “Entrance ticket”, and “Park” emerged with high frequency. Among them, reviews concerning “parking” were mostly negative.
Third, vocabulary related to cultural themes: In the reviews on the Lu’s Salt Merchant Residence, words such as “Huaiyang cuisine”, and “Eat” showed a high frequency; meanwhile, terms including “Garden”, “Rockery” and “Salt merchant” commonly appeared in the word spectrum of all former residences.
Fourth, vocabulary related to exhibition forms and visitor experience: “Take a picture” was a high-frequency behavioral term, while words associated with immersive interaction and in-depth narrative were rarely mentioned in the reviews.

#### Analysis of high-frequency word semantic network.

**Fig 3:**
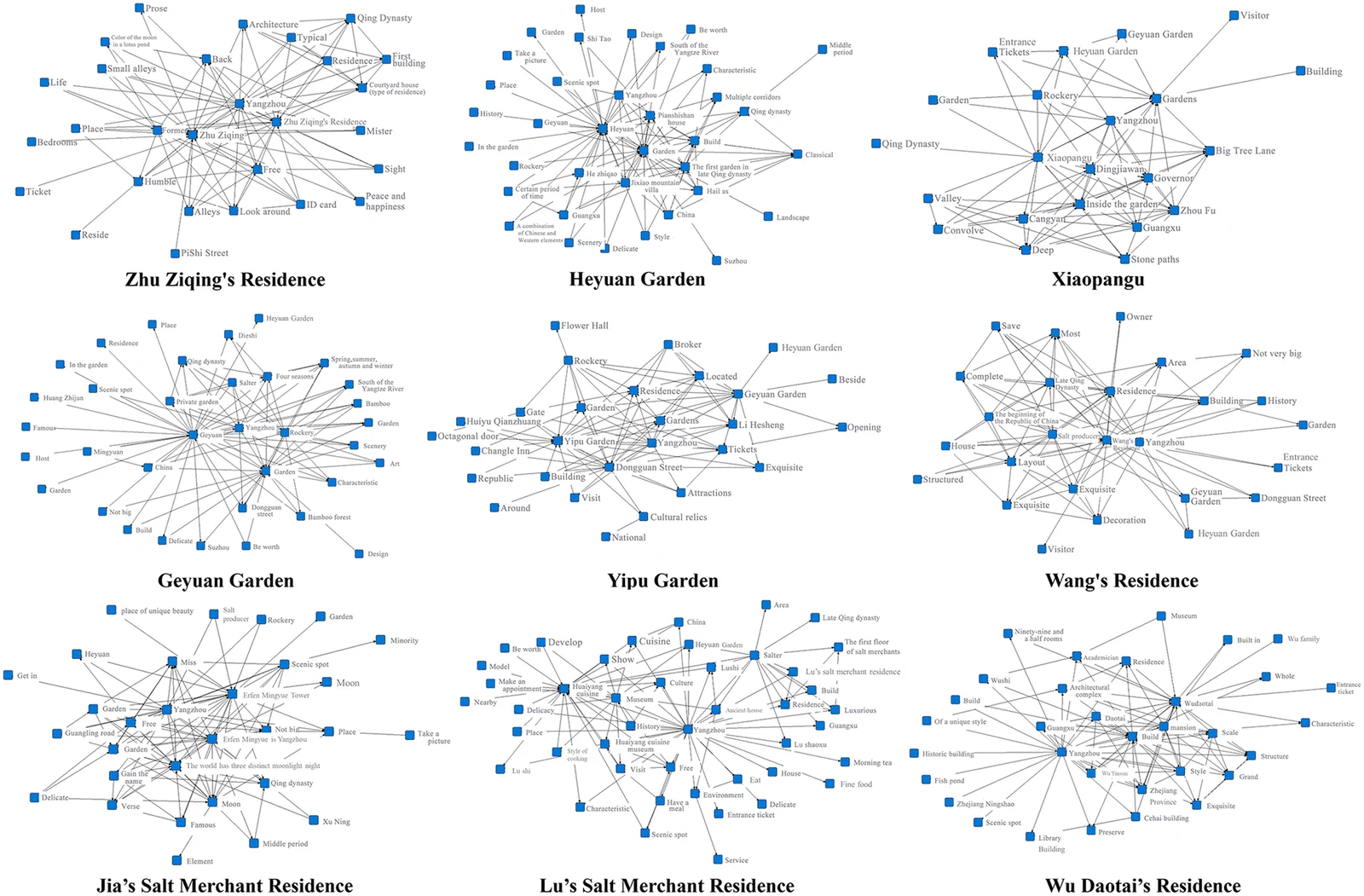
Semantic network analysis of high-frequency words.

The high-frequency word semantic network was constructed in a visual and graphical form, to reveal the semantic relationships, conceptual relevance, and centrality between high-frequency words, thus facilitating a multidimensional understanding of characteristics and semantic structure. In the graph, the more lines connecting nodes, the stronger the correlation. In this study, semantic network analysis was conducted independently for each of 9 former residences. Specifically, a high-frequency word co-occurrence matrix and network diagram (Fig 3) were separately constructed for each residence based on its own review data.

According to the analysis results, the semantic networks of multiple former residences share a highly similar cluster, with words such as “garden”, “rockery”, “piled stones”, and “architecture” forming the center. In multiple semantic networks, the terms “Geyuan Garden” and “Heyuan Garden” show generalized correlations with core words, whereas the names of celebrities such as “Wu Daotai” and “Zhu Ziqing” fail to become core nodes. Words like “photographing” and “visiting” are strongly correlated with landscape vocabulary, while their connections to terms such as “history” and “culture” remain weak. High-frequency words including “small scale” and “admission ticket” appear at the periphery of the network and are only linked to landscape-related terms.

#### Coding results of online negative reviews.

3,922 negative reviews were screened out from the text data in this study. Through initial coding, sub-theme coding and core theme coding, a negative evaluation system containing 5 core themes, 13 sub-themes and several specific manifestations was summarized (Table 3) [61]. The frequencies reported in Table 3 refer to coded mentions rather than unique reviews; therefore, their total may exceed the number of screened negative reviews.

**Table 3:** Negative review coding for former residences of celebrities in Yangzhou.

Core theme (mention frequency)	Sub-theme (mention frequency)	Initial coding (mention frequency)
Accessibility and exhibition design (1241)	Limited accessibility (567)	Few sightseeing attractions (386), limited accessible spaces (158), mismatched publicity (6), being used for offices or commercial purposes (17)
	Monotonous exhibition content (494)	Simple exhibition forms (302), superficial contents (51), limited visiting content (66), short visit duration (75)
	Poor architecture preservation (180)	Dilapidated buildings (98), improper preservation of relics and facilities (73), decaying environment (9)
Management, operation and maintenance (549)	Confused consumption price (259)	High ticket price (175), cash-only payment method (32), confusing prices and lack of public disclosure (27), low cost effectiveness (25)
	Insufficient management (109)	Disorganized management (27), excessive mosquitoes (24), unclear opening hours (58)
	Poor staff attitude and skills (181)	Rude ticket staff (62), rude attitude of staff selling drinks/ice cream (15), short guided tours (28), monotonous guide content (12), lack of guides (53), unprofessional guides (11)
Facility development (444)	Lack of facility maintenance (143)	Insufficient infrastructure (54), inadequate maintenance (67), poor sanitation (20), dirty restroom (2)
	Insufficient transport and surrounding supporting facilities (301)	Difficult to locate (79), lack of signage (42), inaccurate navigation (55), too many bicycles around (23), inconvenient transportation (11), parking difficulties (55), insufficient surrounding facilities (36)
Tourist experience and activity engagement (1079)	Poor environmental atmosphere (115)	Eerie and desolate (63), being fearful when alone (21), oppressive atmosphere (31)
	Unclear distinguishing features of attractions(589)	Excessive commercial development (77), lackluster experience (60), lacking distinct features (284), unfavorable comparison to other former residences of celebrities (67), similar to other attractions (101)
	Lack of interactive experiences (375)	Poor night scenery (23), uninteresting (97), finished after one round (111), read explanatory plaques only (41), boring (103)
Celebrity culture exploration and representation (646)	Insufficient celebrity culture exploration (393)	Lack of cultural connotation (55), no innovative exploration (19), insufficient educational significance (103), limited understanding of celebrities (142), too little information about celebrities (74)
	Limited celebrity matrix effect (253)	Stumbling upon them by chance (24), not well-known (49), no introduction to the residence’s owner (17), obscure attractions (163)

Within the core category of accessibility and exhibition design, the total number of negative mentions reached 1,241. Among them, the sub-theme of “limited accessibility” comprised 567 coded mentions. The primary coding items with the highest frequency were “few sightseeing attractions” (386 coded mentions) and “limited accessible spaces” (158 coded mentions). The sub-theme of “monotonous exhibition content” comprised 494 coded mentions, dominated by “simple exhibition forms” (302 coded mentions), followed by “short visit duration” (75 coded mentions) and “limited visiting content” (66 coded mentions). The sub-theme of “poor architecture preservation” comprised 180 coded mentions, mainly including “dilapidated buildings” (98 coded mentions) and “improper preservation of relics and facilities” (73 coded mentions). The data indicate that tourists’ complaints are most concentrated on the scale of exhibition and the monotony of display content.

Within the core category of management, operation and maintenance, the total number of negative mentions reached 549. The sub-theme of “confused consumption price” appeared 259 coded mentions, with “high ticket price” (175 coded mentions) as the main complaint, followed by “cash-only payment method” (32 coded mentions). Under the theme of “poor staff attitude and skills” (181 coded mentions), “rude ticket staff” (62 coded mentions) and “lack of guides” (53 coded mentions) registered relatively high frequencies. The sub-theme of “inadequate management” appeared 109 coded mentions, among which the issue of “unclear opening hours” (58 coded mentions) was particularly prominent.

Within the core category of facility development, the total number of negative mentions reached 444. The sub-theme of “insufficient transport and surrounding supporting facilities” appeared 301 coded mentions, with high-frequency problems including “difficult to locate” (79 coded mentions), “inaccurate navigation” (55 coded mentions), “parking difficulties” (55 coded mentions), and “lack of signage” (42 coded mentions). The sub-theme of “lack of facility maintenance” appeared 143 coded mentions, mainly reflected in “inadequate maintenance” (67 coded mentions) and “insufficient infrastructure” (54 coded mentions). It is evident that tourists complained far more about the accessibility and surrounding supporting facilities of the former residences than about the maintenance of internal facilities.

Within the core category of tourist experience and activity engagement, the total number of negative mentions reached 1,079. The sub-theme of “unclear distinguishing features of attractions” appeared 589 coded mentions, among which “lacking distinct features” (284 coded mentions) and “similar to other attractions” (101 coded mentions) had the highest frequencies. The sub-theme of “lack of interactive experiences” appeared 375 coded mentions, mainly manifested as one simple tour with “finished after one round” (111 coded mentions) and “boring” (103 coded mentions). The sub-theme of “poor environmental atmosphere” appeared 115 coded mentions, mainly reflected in “eerie and desolate” (63 coded mentions) and “oppressive atmosphere” (31 coded mentions). These data reflect tourists’ strong dissatisfaction with the homogenization of tourism experience and the lack of interactive participation.

Within the core category of celebrity culture exploration and representation, the total number of negative mentions reached 646. The sub-theme of “insufficient celebrity culture exploration” appeared 393 coded mentions, with the core complaints being “limited understanding of celebrities” (142 coded mentions) and “insufficient educational significance” (103 coded mentions). The sub-theme of “limited celebrity matrix effect” appeared 253 coded mentions, mainly reflected in “obscure attractions” (163 coded mentions) and “not well-known” (49 coded mentions). This indicates that tourists generally perceive that the cultural connotations of historical celebrities have not been conveyed, and most former residences enjoy low popularity.

### Questionnaire survey results regarding former residences of celebrities in Yangzhou

Based on the preliminary online review analysis results, this study established the hierarchical structure and specific elements of the POE for former residences of celebrities in Yangzhou, consisting of 5 major dimensions and 28 evaluation elements. Satisfaction surveys were conducted among users regarding attraction development, service and management, environment and facilities, atmosphere and activities, and characteristics and exhibition of celebrity culture. Cronbach’s α coefficient was used to test the reliability of the questionnaire. Overall, the Cronbach’s α coefficient of the entire questionnaire was 0.892, higher than the threshold of 0.8, indicating good questionnaire reliability. Exploratory factor analysis was adopted for validity testing. In case of KMO = 0.891, and Bartlett test *p* < 0.001, the questionnaire possesses good structural validity.

The questionnaire results were further analyzed. Among the 1,643 respondents, the proportion of males and females was basically balanced. Respondents aged 18–45 constituted the main group, accounting for 76.8% of all participants. Participants with college/bachelor’s degree or above accounts for 71.5%, and first-time visitors account for 63.2%. Overall, the sample structure is relatively balanced and presents good representativeness (Table 4).

**Table 4:** Demographic characteristics of respondents.

Category		Frequency	Percentage (%)
**Gender**	Male	744	45.3
	Female	899	54.7
**Age**	Below 18 years old	82	5.0
	18-30 years old	468	28.5
	31-45 years old	793	48.3
	46-60 years old	246	15.0
	Over 60 years old	54	3.3
**Education background**	Junior high school and below	164	10.0
	High school/technical secondary school	304	18.5
	College/bachelor’s degree	1025	62.4
	Master or above	150	9.1
**Visiting frequency**	First visit	1,038	63.2
	Second visit and above	605	36.8
**Number of former residences visited (this trip)**	1	586	35.7
	2-3 sites	804	48.9
	4 sites and above	253	15.4

Note: Percentages are rounded to one decimal place; therefore, totals may not sum exactly to 100.0%.

Overall, the total tourism evaluation for the former residences of celebrities in Yangzhou was 61.56 points (Table 5). While these residences have demonstrated certain strengths in attraction development and service management, they received lower ratings in environmental facilities, atmosphere creation, and presentation of celebrity culture. Further improvements and refinements are needed.

**Table 5:** Evaluation results of former residences of celebrities in Yangzhou.

Target layer	Score	Criterion layer	Weight	Score	Sub-criterion layer	Weight	Score
Evaluation of former residences of celebrities in Yangzhou	61.56	Attraction development	0.25201	70.33	Attractiveness	0.14325	69.35
					Spatial planning	0.14187	68.54
					Function utilization	0.13849	66.24
					Exhibition content and form	0.10278	59.32
					Architecture preservation	0.24127	79.82
					Preservation of interior artifacts	0.11171	61.11
					External environmental development	0.12063	77.21
		Service and management	0.16767	68.05	Consumption costs and payment methods	0.23890	59.61
					Consumer safety	0.09264	81.21
					Management practices	0.19811	70.14
					Staff attitude and professional level	0.16036	71.23
					Guide service	0.19811	68.22
					Online service	0.11188	66.63
		Environment and facilities	0.12729	56.70	Infrastructure	0.11483	71.11
					Signage installation	0.13551	51.08
					Attraction accessibility	0.31335	50.22
					Parking facilities	0.16748	53.31
					Surrounding supporting facilities	0.14444	52.27
					Hygiene	0.12440	75.55
		Atmosphere and activities	0.16767	53.05	Environmental atmosphere	0.18602	62.24
					Fun and interactivity	0.21602	49.97
					Activity diversity	0.24760	48.80
					Night tour programs	0.10275	57.99
					Development of unique advantages	0.24760	51.02
		Characteristics and exhibition of celebrity culture	0.28535	57.16	Demonstration of cultural-educational value	0.33333	57.13
					Innovative expression methods	0.16667	50.47
					Characteristics of celebrities and exhibition forms	0.33333	58.97
					Promotion and recognition of celebrity culture	0.16667	60.29

### Attraction development

This dimension scores 70.33 points, ranking the highest among the five criterion layers. Among the sub-indicators, architecture preservation (79.82 points) and external environmental development (77.21 points) score markedly higher than other items. By contrast, exhibition content and form (59.32 points) and preservation of interior artifacts (61.11 points) obtain relatively low scores.

### Service and management

The overall score of this dimension is 68.05 points. At the sub-criterion layer, consumer safety (81.21 points) and hygiene (75.55 points) achieve relatively high scores. Consumption costs and payment methods score the lowest (59.61 points), showing a conspicuous gap compared with other sub-criterion indicators.

### Environment and facilities

This dimension gains 56.70 points, ranking second lowest among the five criterion layers. Scores of all sub-criterion indicators are below 76 points. Among them, signage installation (51.08 points) and attraction accessibility (50.22 points) are the two lowest-scoring indicators within this dimension; meanwhile, parking facilities (53.31 points) and surrounding supporting facilities (52.27 points) also remain at a low level.

### Atmosphere and activities

The overall score of this dimension is 53.05 points. At the sub‑criterion layer, environmental atmosphere ranks the highest (62.24 points), followed by night tour programs (57.99 points). By contrast, fun and interactivity (49.97 points), activity diversity (48.80 points), and development of unique advantages (51.02 points) are the three lowest‑scoring indicators, with the last of these reflecting the insufficient development of distinctive features across the former residences.

### Characteristics and exhibition of celebrity culture

The overall score of this dimension is 57.16 points. Scores of sub‑criterion layers in descending order are as follows: promotion and recognition of celebrity culture (60.29 points), characteristics of celebrities and exhibition forms (58.97 points), demonstration of cultural-educational value (57.13 points), and innovative expression methods (50.47 points). The score of innovative expression methods is markedly lower than that of the other three items.

### Comparison between online negative review coding system and questionnaire survey

Given that the aggregate categories derived from online reviews and the questionnaire evaluation dimensions differ in the level of comparison, direct pairwise comparison at the macro level cannot accurately reflect the partial correspondence between the two datasets. Therefore, this study focuses the comparison on the sub-dimension level to examine the partial correspondence in identifying specific problems.

At the sub-dimension layer, the alignment between the two datasets is evident:

In the questionnaire, “signage installation” (51.08 points), “attraction accessibility” (50.22 points), and “parking facilities” (53.31 points) are the three lowest-scoring indicators within the environment and facilities dimension. In online coding, the category “insufficient transport and surrounding supporting facilities” appears 301 coded mentions, including 79 coded mentions of “difficult to locate”, 42 coded mentions of “lack of signage”, and 55 coded mentions of “parking difficulties”.

In the questionnaire, the scores for “fun and interactivity” (49.97 points), “activity diversity” (48.80 points), and “development of unique advantages” (51.02 points) are low. In the online content coding results, there are 375 coded mentions of “lack of interactive experiences” and 494 coded mentions of “monotonous exhibition content”, including 111 coded mentions of “finished after one round” and 103 coded mentions of “boring”.

In the questionnaire, “innovative expression methods” (50.47 points) is relatively low; in the online coding results, there are 393 coded mentions of “insufficient celebrity culture exploration” and 589 coded mentions of “unclear distinguishing features of attractions”, including 142 coded mentions of “limited understanding of celebrities” and 284 coded mentions of “lacking distinct features”.

In the questionnaire, indicators including “architecture preservation” (79.82 points), “external environmental development” (77.21 points), “consumer safety” (81.21 points), “management practices” (70.14 points), “infrastructure” (71.11 points), and “hygiene” (75.55 points) all score above 70 points, indicating a high overall level of tourist satisfaction with these aspects; correspondingly, the mention frequencies of such categories in online negative review are also relatively low.

Discrepancies exist between the two types of data across certain dimensions. For example, at the core category level, ‘accessibility and exhibition design’ receives the highest frequency of negative mentions (1,241 coded mentions), yet its corresponding questionnaire dimension, ‘attraction development,’ achieves a relatively high score (70.33 points). This apparent reverse pairing is further examined in the Discussion, where its underlying causes are analyzed. At the sub‑dimension level, online negative reviews contain a considerable number of coded mentions regarding ‘poor staff attitude and skills’ (181 coded mentions), whereas the questionnaire score for ‘staff attitude and professional level’ reaches 71.23 points (above 70 points).

## Discussion

### Core problems and data complementation mechanism of post-occupancy evaluation of former residences of celebrities in Yangzhou

Adopting a sequential mixed-methods design, this study conducts a multi-dimensional evaluation on the same set of research issues through two stages: exploration based on online review data and quantitative assessment via questionnaire data. The findings reveal that the post-occupancy evaluation of 9 former residences of celebrities in Yangzhou presents the following characteristics:

First, weak cultural expression and insufficient interactive experience. Tourists’ perception of former residences of celebrities in Yangzhou presents a high degree of homogenization, making it difficult to distinguish the unique cultural characteristics of different celebrities. Various former residences are generally categorized into generalized concepts such as “Yangzhou Gardens” and “Salt Merchants”. Meanwhile, these former residences lack innovative expression methods in cultural display, failing to convey the life stories and spiritual connotations of historical celebrities. In addition, the tourist experience tends to be superficial and monotonous in mode. Cultural heritage is a socially constructed cultural process [62]. The officially recognized authoritative heritage discourse usually focuses on the expression of physical form, chronological value and architectural style, while marginalizing the localized, vivid memories and narratives associated with specific historical figures. At present, the construction and promotion of former residences of celebrities in Yangzhou follow the official heritage discourse centered on “garden architecture”, rather than popular narratives focused on “celebrity stories”. This results in tourists being unable to perceive the unique culture of individual celebrities and leads to homogenized public cognition. Meanwhile, as public spaces, the social value of such cultural heritage stems from the positive interaction between ordinary people’s daily cultural participation and institutional management, namely the experience and interpretation of cultural heritage [63]. In this study, such interactive mechanism is absent in former residences of celebrities in Yangzhou. Online reviews such as “finished after one round” and “limited understanding of celebrities” indicate that these sites merely conform to the rules of authoritative heritage discourse while losing their public attributes. Insufficient attention to public participation weakens the popular narrative of celebrity culture, resulting in poor experiential and interactive performance. Some literature regards the city as an “institution”, and defines the “core business” of heritage site management as maintaining and attracting “ideal citizens” and promoting economic growth [64]. Under this logic, if former residences of celebrities cannot serve the goals of “attracting tourists” and “driving economic growth”, their in-depth historical narratives may be sacrificed and reduced to a generalized backdrop. This aligns with Cremaschi and Vitale’s argument that historic spaces are employed as tools to achieve specific policy objectives, rather than to shape public cultural practices [65].

Second, inadequate accessibility and signage system. Tourists face prominent difficulties in reaching the former residences due to inadequate signage systems and inaccurate navigation information, making it difficult for them to locate the attraction smoothly. Essentially, the accessibility problem arises because the development of former residences of celebrities prioritizes commercialization and complies with official conservation planning requirements to preserve the original pattern of alleys in the ancient urban area. It neglects the urban infrastructure construction of signage systems and humanistic care practices, and lacks the construction of inclusive public realms. When the signage system lacks entertainment appeal and attractiveness, the process of route finding becomes tedious, which further undermines tourists’ overall satisfaction with the visit [66]. Therefore, insufficient accessibility is not merely a problem of planning and design, but a manifestation of conflicts in governance logic.

Third, excessive commercialization and unbalanced resource allocation. In online reviews on the Lu’s Salt Merchant Residence, commercial terms such as “restaurant”, “Huaiyang cuisine” and “dining” appear far more frequently than cultural terms. By contrast, though Heyuan Garden and Geyuan Garden are also equipped with commercial facilities, cultural vocabulary still predominates, with far fewer negative comments. Public spaces are confronted with an inherent conflict between commodified and non-commodified usage. Cultural experience and historical ambiance are replaced by commercial scenarios, giving rise to public complaints about “the encroachment upon public resources”. Dissatisfaction with the erosion of historical ambiance caused by “excessive commercial development” reflects the tension between commodified and non-commodified institutional arrangements for public space use, as theorized by Burini [67]. Publicly accessible urban spaces function as public services. The governance evaluation of such spaces should examine not only their physical infrastructure but also their capacity to sustain conviviality and a sense of civic belonging [68].

In addition, Heyuan Garden and Geyuan Garden account for the vast majority of tourist reviews, while former residences such as Zhu Ziqing’s Residence and Wang’s Residence are labeled as “obscure attractions” or “stumbling upon them by chance”, with insufficient development of their own distinctive advantages. Official conservation and promotional resources tend to be concentrated on a small number of “canonized” heritage sites [69], whereas other former residences with prominent celebrity cultural value are marginalized, resulting in severely unbalanced public perception and low overall utilization rate of cultural heritage resources. To a certain extent, this also reflects that the development of urban cultural heritage is not oriented to public needs or popular culture, but serves official governance priorities. It merely highlights individual characteristic landmarks rather than attaching importance to holistic development [70].

Fourth, some sites receive numerous negative online reviews yet achieve acceptable questionnaire scores. For instance, “accessibility and exhibition design” receives the highest frequency of negative mentions (1,241 coded mentions) among online reviews, yet its corresponding questionnaire dimension, “attraction development” scores the highest among the five dimensions (70.33 points). This reverse pairing appears contradictory on the surface, but is in fact the result of internal polarization within this category. “accessibility and exhibition design” encompasses seven sub‑indicators, among which “architecture preservation” (79.82 points) and “external environmental development” (77.21 points) receive relatively high scores, while the remaining sub‑indicators score lower. The high‑scoring items pull up the overall average, resulting in a relatively high composite score at the questionnaire level. The high frequency of negative online mentions is also mainly concentrated on the limited accessibility or monotonous exhibition content, such as “monotonous exhibition content” and “few sightseeing attractions”. The granularity difference between the two types of data reflects that online reviews excel at pinpointing specific shortcomings, while questionnaire data captures tourists’ overall perception of a given category. When a “ranking mismatch” occurs, it does not indicate contradiction between the datasets, but rather reveals the structural differentiation within the same category, where strengths and weaknesses coexist, and each is highlighted differently depending on the data form.

Furthermore, online reviews contain many complaints about staff service attitude and professional competence, while the overall satisfaction rating still remains at a good level. This discrepancy is consistent with classic findings in online review research. People are far more motivated to post negative reviews than neutral or positive ones. Tourists with poor experiences are more inclined to write reviews than those with moderate experiences [37], resulting in a “J-shaped” or “inverted L-shaped” review distribution (with a large number of positive and negative but few moderate ones in the middle) [71]. Furthermore, negative reviews tend to exert a greater influence on public decision-making than positive ones. They are also less susceptible to manipulation by stakeholders and therefore possess higher credibility [72]. Accordingly, the negative bias observed in this study should not be regarded merely as isolated cases, but as a reflection of localized pain points.

As digital trace data, online reviews face unique threats to their validity. For example, the sample is non-random (only a portion of users post reviews), user behavior is affected by platform incentive mechanisms, and algorithmic filtering leads to bias in visible content [41]. To mitigate these threats, this study adopts a hybrid strategy “dominated by manual identification and supplemented by automatic screening”, and tests the stability of core conclusions through hierarchical platform analysis. Even so, self-selection bias among reviewers and the potential impact of platform review policies cannot be completely eliminated, which readers should bear in mind when interpreting the results. The online data we observe does not represent the full picture, but has been filtered and ranked by platform algorithms [73]. Platforms prioritize displaying sensational and eye-catching extreme content, which further distorts the data. Many reviews selected in this study are partial and isolated evaluations, such as complaints about “rude attitude of staff selling drinks/ice cream”. By contrast, the questionnaire requires tourists to rate holistic concepts such as “staff attitude and professional level”. When scoring, tourists tend to make an overall average assessment subconsciously. They think that “although a few staff members have poor attitudes, the overall service is decent”, thereby weakening the impact of individual negative cases. In the anonymous online environment, tourists are more inclined to criticize with stronger tone and are more likely to magnify negative feelings [74,75]. The questionnaire results reveal relatively high scores for these items, indicating that former residences of celebrities in Yangzhou have basically met tourists’ basic acceptance standards in these fundamental aspects, with no systematic deficiencies observed. The divergence between the two types of data does not imply a simple right-or-wrong distinction; instead, each reflects social reality at different levels. Online review data captures extreme individual cases, reminding managers that even when overall performance reaches the standard, localized shortcomings still remain to be addressed. By contrast, questionnaire data reflects the overall evaluation distribution. This mechanism is proposed as an explanatory hypothesis in this study. Due to data constraints, a systematic comparison of algorithmic amplification effects across different platforms has not been conducted, leaving this mechanism to be further validated in subsequent research.

Overall, online evaluation data features a large sample size and easy accessibility. Its openness allows tourists to freely raise any issues of concern, helping researchers identify previously unconsidered factors and avoid research blind spots. Such data is effective in discovering, early-warning, and tracking both macroscopic trends and specific microscopic problems, reflecting the public’s immediate, spontaneous, and emotional genuine perceptions [76]. Meanwhile, online data reveals detailed pain points that traditional questionnaires may overlook. It excels at capturing specific problem details and genuine emotional perceptions, though review content tends to be skewed toward negative sentiment. For example, tourists may feel frightened simply because there are too few visitors around, which is not caused by the former residences of celebrities themselves. It is evident that online review data is susceptible to subjective factors and potential exaggeration, while also exhibiting inherent uncontrollability and randomness. Traditional questionnaire data is limited in sample size, yet it excels at quantifying the prevalence and relative weight of problems, facilitating in-depth professional analysis and the identification of targeted issues; however, it struggles to present the detailed causes of problems and their emotional contexts. The integration of the two types of data can not only leverage the high granularity of online data to compensate for the preset limitations of questionnaires, but also utilize the quantifiable nature of questionnaire data to correct the sample bias inherent in online data, which reduces research errors caused by one-sided data sources.

It should be noted that the questionnaire evaluation indicators in this study were constructed based on the negative coding results of online reviews. Therefore, the correspondence between the two types of data in diagnosing core problems is, to a certain extent, an expected outcome of the research design itself, rather than a validation between two completely independent tools. This study does not aim to demonstrate that the two types of data mutually validate each other, but rather to explore their complementarity. Online reviews excel at providing specific details and contextual descriptions, while questionnaire data are good at measuring the prevalence and relative weight of problems. Meanwhile, the discrepancies between the two types of data also offer meaningful insights, revealing the difference between extreme individual cases and overall public perception.

### Theoretical implications for urban governance and heritage research

This study reveals that the development of former residences of celebrities in Yangzhou represents a game between cultural commodification and public participation. Similar to the governance of other historic urban spaces, its management is also shaped by the need to balance the preservation of celebrity cultural memory, tourists’ experiential needs, and tourism economic benefits. This is not merely a technical or administrative issue; instead, it is driven simultaneously by three distinct logics: symbolic logic, civic logic, and administrative logic, which often conflict with one another and create inherent tensions [66]. A single research method cannot capture the multiple dimensions of these tensions simultaneously, whereas the adoption of mixed methods enables core problems to be identified through convergent identification. This provides a more comprehensive diagnostic framework for the governance of cultural heritage and even historic urban spaces.

Cultural heritage management authorities can adopt the “online data + traditional survey” approach proposed in this study to establish a complementary mechanism for information collection and analysis, and integrate the regular monitoring of online reviews with periodic structured satisfaction surveys to jointly support administrative decision-making. Online review data can identify specific issues not predefined by researchers, provide tourists’ genuine emotions and detailed descriptions, reveal systematic defects, and give early warnings of potential conflicts. For instance, online reviews refine the general problem of “insufficient signage” into concrete complaints such as “ineffective navigation” and “inadequate signage”. Traditional questionnaire surveys can assess the prevalence and severity of existing problems, calculate the contribution weight of each dimension to overall satisfaction, provide a basis for prioritizing resource allocation, and rectify emotional amplification and self-selection bias inherent in online data. For example, questionnaire data indicates that accessibility and cultural experience constitute the core shortcomings with the lowest evaluation scores. Although ticket prices are frequently criticized online, their actual influence weight is lower than the former two. Problems identified simultaneously by both datasets should be prioritized for improvement. Issues that appear only in online reviews but do not show significantly low scores in questionnaires are likely situational or individual emotional responses.

In practical operation, online data can serve as a source of daily early warning. Discover new specific pain points in real time and achieve rapid response by continuously capturing online reviews. Questionnaire data can function as a periodic diagnostic tool. A structured satisfaction survey should be conducted once every one or two years to quantify the weight and satisfaction score of each dimension, identify systematic shortcomings, and guide medium- and long-term resource allocation. This differentiated approach helps managers avoid being misled by extreme reviews while not ignoring objectively existing systematic defects. When the same type of problem recurs frequently in online reviews, a dedicated questionnaire module can be launched to assess its prevalence and weight. Conversely, dimensions with low questionnaire scores can be traced through online reviews to explore their specific manifestations and emotional contexts. This paradigm can be widely applied to cultural heritage sites of different types and in various regions. It emphasizes integrating public evaluation into heritage site management and provides an operational tool for understanding the relationships among cultural commodification, public participation and local identity.

### Future development strategies

Tourists generally complain about few sightseeing attractions and limited accessible spaces, noting a notable gap between the actual touring area and their expectations. Meanwhile, the exhibition arrangement is simplistic with superficial content, lacking depth and appeal. Under the premise of heritage conservation, appropriately expand the open areas, and renovate part of the idle spaces into interactive experience zones, rest areas or temporary exhibition halls. Excavate historical stories of notable figures, and enhance exhibition appeal through digital story walls, immersive short films and other forms. In view of dilapidated buildings and degraded environments, a multidisciplinary team should be organized to conduct scientific restoration based on the principle of “restoring the old as it was”, and establish a long-term monitoring and preventive conservation mechanism.

Tourists have reflected high ticket price, cash-only payment method in some attractions, poor staff attitude, short guided tours and monotonous guide content. It is suggested to reassess ticket pricing, implement differentiated ticket fares, and fully roll out electronic payment and online ticket booking services. Establish standardized service training and assessment mechanisms to improve the professional competence of staff. Provide professional training in historical and cultural knowledge for tour guides, and launch intelligent audio guides as a supplementary service. Meanwhile, clearly publicize opening hours, conduct regular pest elimination, and strengthen daily O&&M management.

Tourists generally report deficiencies in the signage system, inaccurate navigation, and parking difficulties, as well as a lack of supporting facilities such as surrounding commercial services and rest areas. It is recommended to install clear signposts at major intersections around the former residences to form a continuous guidance route, and cooperate with mainstream map APPs to optimize navigation and positioning. Utilize the limited surrounding space to plan multi-story parking lots or time-sharing shared parking spaces, and set up temporary parking areas and shuttle buses during peak tourist hours. Ensure sufficient public restrooms, seating benches and drinking water facilities, and rationally plan supporting commercial facilities with local cultural features such as catering outlets and souvenir shops in the surrounding area.

Tourists argue that the cultural narrative of the exhibitions is superficial, failing to thoroughly explore the celebrities’ life stories, spiritual connotations and historical backgrounds; most former residences remain low in popularity and have not developed distinctive celebrity cultural IPs. It is suggested to collaborate with universities and research institutions to further explore celebrity culture and its historical connections with Yangzhou, and transform academic findings into accessible exhibition narratives and storylines for the public. Link the scattered former residences of historical figures into themed tourist routes, launch combined tickets, strengthen integrated marketing, and generate scale effects. Each residence should define its core cultural positioning. For example, Zhu Ziqing’s Residence features a “literature-themed tour”, while salt merchant mansions focus on “salt merchant culture”, so as to avoid homogenization.

Some former residences present a desolate and somber atmosphere with a monotonous sightseeing mode and a scarcity of interactive experiences. It is advisable to optimize landscape lighting, add rest facilities and green landscapes with historical charm, and build a pleasant ambient environment. Install low-cost interactive facilities such as touch screens and souvenir stamp check-in points to enhance tourists’ sense of engagement.

### Limitations

First, online evaluations encompass multi-form data including texts, images and videos. This study only selects textual information from several mainstream websites as research samples, with limited data sources. In addition, the volume of textual reviews varies across different former residences of celebrities. Although the semantic network analysis in this study is conducted independently, and the core conclusions are mainly based on the common patterns across different residences, which are not affected by individual fluctuations of residences with small sample sizes. For former residences with an excessively low number of reviews, such as Yipu Garden, the co-occurrence matrix constructed merely based on 264 reviews may be relatively sparse, resulting in poor stability of the network structure. Be cautious in interpreting its node relationships. In future research, the number of research objects can be further expanded by incorporating more former residences of celebrities with abundant online reviews into the research scope; meanwhile, more diversified research data such as tourist ratings, travel photos and travel videos can be adopted. It is necessary to further explore effective methods for integrating these different data types, compensate for the analytical deficiencies of former residences lacking sufficient textual reviews, and ensure higher accuracy of research findings.

Second, given that online review data cannot capture tourists’ detailed personal information [41], it is difficult to explore the evaluation differences among tourists of varying age, gender, education level and other demographic characteristics towards former residences of celebrities in Yangzhou. Subsequent studies can further adopt research methods such as offline interviews and field observation, so as to explore the needs of different types of tourists for former residences of celebrities in Yangzhou.

Third, this study mainly focuses on identifying the key deficiencies of former residences of celebrities in Yangzhou, so the analysis centers on negative reviews without systematically incorporating positive or neutral ones. Such a targeted focus serves clear research purposes yet may introduce two types of bias. First, tourists with extremely poor experiences are more inclined to post reviews than satisfied visitors, which may lead to an overestimation of the frequency of certain problems. Second, the advantageous reflected in positive reviews are excluded from the negative coding system, which may render problem diagnosis overly prominent while overlooking existing successful practices. Future research may adopt sentiment-balanced sampling or conduct supplementary coding of positive and neutral reviews, so as to construct a comprehensive evaluation system covering both strengths and deficiencies and derive more balanced diagnostic conclusions.

Fourth, the online reviews in this study cover a long-time span, while the distribution of offline questionnaire collection is concentrated in winter with a limited duration. To assess the potential influence of seasonal factors on the findings, this study conducted a chi‑square test on the distribution of core negative categories in online reviews between winter and non‑winter periods (see Table S3-1 in S3 File). The results show that *χ²(4)* ≈ 0.19, *p* ≈ 0.996, indicating no significant difference in the distribution of core negative themes between winter (December-February) and non-winter(March-November), suggesting that seasonal factors exert limited structural influence on the core evaluation dimensions.

Nevertheless, we acknowledge that the offline data only reflects the perceptions of winter tourists and may fail to capture season-specific experiential dimensions such as spring floral landscapes and summer retreat needs. It is recommended that future researches supplement questionnaire data in spring, summer and autumn to verify the year-round applicability of the conclusions drawn in this study.

Fifth, as Yangzhou is a popular tourist city, the international tourism boom has gradually recovered with the easing of the pandemic. Future research may further collect review data from international tourism websites to expand the existing researches, and analyze the development of Chinese celebrity culture in international tourism, which is conducive to broadening the international influence of culture with Chinese characteristics.

## Conclusions

Taking 9 national-level former residences of celebrities in Yangzhou as cases, this study adopts a sequential mixed-methods design and integrates online review data with offline questionnaire data. The findings reveal that, in diagnosing core shortcomings, the two types of data jointly point to issues such as weak cultural expression, poor accessibility, excessive commercialization, lack of interactive experiences, and uneven resource distribution. The two types of data can achieve functional complementarity. Online data excels at identifying specific problems and capturing public emotional perceptions, while questionnaire data is good at quantifying problem weights and assessing their prevalence. The integration of the two forms a complementary chain of “problem identification - weight quantification - detail refinement”.

Based on the analysis results, this study suggests that cultural heritage management authorities establish a complementary mechanism combining online data and traditional surveys. Taking online data as a daily early warning tool and questionnaire data as a periodic diagnostic tool, the mechanism jointly supports management decision-making and provides an operable mixed-methods paradigm for the targeted governance of cultural heritage. Future research can expand data sources and collection types, supplement multi-seasonal survey data, and broaden international perspectives to verify the robustness of the research conclusions.

## Supporting information

S1 Table Extraction of high-frequency words. (DOCX)

S1 File AHP-FCE evaluation model, data and calculation results. (DOCX)

S2 File Post-occupancy Evaluation (POE) questionnaire for former residences of celebrities in Yangzhou. (DOCX)

S3 File Comparison of occurrence frequencies of online review categories between winter and non‑winter. (DOCX)
