# Supplementary material for: Post-occupancy evaluation of former residences of celebrities in Yangzhou based on the comparison of online reviews and questionnaire data
Source: PLoS One. 2026 Sep 3;21(9):e0357793. doi: 10.1371/journal.pone.0357793 (PMC13541147; doi:10.1371/journal.pone.0357793)
Supplement: Supplementary file 1 [file pone.0357793.s001.docx]

**Table1 Extraction of high-frequency words**

| **Xiaopangu** | | **Jia’s Salt Merchant Residence** | | **Yipu Garden** | |
| --- | --- | --- | --- | --- | --- |
| **Words and Expressions** | **Frequency** | **Words and Expressions** | **Frequency** | **Words and Expressions** | **Frequency** |
| Xiaopangu | 181 | Erfen Mingyue Tower | 373 | Yipu Garden | 210 |
| Yangzhou | 139 | Yangzhou | 357 | Yangzhou | 145 |
| Garden | 136 | Moon | 249 | Dongguan street | 141 |
| Rockery | 97 | Garden | 216 | Geyuan Garden | 110 |
| Courtyard | 85 | Erfen Mingyue is Yangzhou | 182 | Garden | 96 |
| Heyuan Garden | 68 | The world’s three shares of moonlit night (Note: This is a famous line from a poem by Xu Ning of Tang Dynasty) | 177 | Entrance ticket | 78 |
| Entrance ticket | 59 | Free | 171 | Courtyard | 73 |
| Locality | 45 | Locality | 149 | Rockery | 67 |
| DingJiaWan | 43 | Take a picture | 133 | Building | 41 |
| Garden | 43 | Not often | 115 | Visit | 36 |
| feature spot | 43 | Don’t have | 100 | Li Hesheng | 36 |
| Visitor | 42 | Heyuan Garden | 100 | Come into | 33 |
| Zhou Fu | 41 | Courtyard | 96 | Locality | 32 |
| Park | 38 | Guangling road | 94 | The Republic of China | 29 |
| Lane | 36 | Fine | 87 | Garden | 28 |
| Building | 31 | Entrance ticket | 84 | Changle Inn | 28 |
| Nominate | 30 | Geyuan Garden | 77 | Fine | 27 |
| Liangjiang governor | 30 | Rockery | 77 | Cultural relic | 27 |
| Guangxu | 30 | Entrance | 74 | Financial industry | 26 |
| Not often | 29 | Salt merchant | 73 | Broker | 26 |
| **Lu’s Salt Merchant Residence** | | **Wang’s Residence** | | **Geyuan Garden** | |
| **Words and Expressions** | **Frequency** | **Words and Expressions** | **Frequency** | **Words and Expressions** | **Frequency** |
| Yangzhou | 351 | Wang’s Residence | 475 | Geyuan Garden | 1878 |
| Huaiyang cuisine | 313 | Yangzhou | 428 | Garden | 1409 |
| Museum | 172 | Salt merchant | 319 | Yangzhou | 860 |
| Salt merchant | 156 | Geyuan Garden | 306 | Rockery | 765 |
| Huaiyang Cuisine Museum | 140 | House | 267 | Be worth | 711 |
| Lu shi | 138 | Building | 236 | The four seasons | 458 |
| Ancient house | 119 | Heyuan Garden | 160 | Courtyard | 380 |
| Culture | 110 | Garden | 152 | Dongguan street | 378 |
| Visit | 104 | Entrance ticket | 134 | Characteristic | 352 |
| Environment | 101 | Not often | 127 | Regions south of the Yangtze River | 296 |
| History | 92 | Dongguan street | 123 | Salt merchant | 286 |
| Free | 90 | Master | 109 | Locality | 259 |
| Lu’s Salt Merchant Residence | 82 | Locality | 103 | Convenient | 236 |
| Heyuan Garden | 82 | Preserve | 92 | Building | 234 |
| Locality | 77 | Area | 92 | Explain | 227 |
| Eat | 72 | Courtyard | 91 | China | 222 |
| Building | 67 | History | 91 | House | 202 |
| Fine food | 63 | Be worth | 91 | Private | 197 |
| Characteristic | 62 | Visitor | 88 | Not often | 191 |
| Style of cooking | 54 | Garden | 88 | Heyuan Garden | 183 |
| **Heyuan Garden** | | **Zhu Ziqing’s Residence** | | **Wu Daotai’s Residence** | |
| **Words and Expressions** | **Frequency** | **Words and Expressions** | **Frequency** | **Words and Expressions** | **Frequency** |
| Heyuan Garden | 2103 | Zhu Ziqing | 827 | Yangzhou | 377 |
| Garden | 1674 | Yangzhou | 704 | Wu Daotai | 320 |
| Yangzhou | 934 | Zhu Ziqing’s former residence | 523 | Building | 298 |
| Building | 754 | Teacher | 340 | Academician | 141 |
| Be worth | 698 | Visit | 271 | Taotai | 137 |
| Geyuan Garden | 534 | A view of sb.s back | 271 | Style | 96 |
| The first garden in the late Qing Dynasty | 504 | Free | 235 | Wu family | 96 |
| Courtyard | 467 | Locality | 207 | Zhejiang Province | 93 |
| Characteristic | 366 | Not often | 186 | Entrance ticket | 92 |
| Regions south of the Yangtze River | 317 | Certificate of identification | 150 | Wu Yinsun | 88 |
| Locality | 302 | Moonlight over the lotus pond | 134 | Cehai building | 84 |
| China | 299 | All one’s life | 129 | Come into | 74 |
| Stone mountain room | 282 | Three-section compound | 121 | Jiangnan three famous house | 73 |
| Style | 279 | Pishi street | 120 | Guangxu | 73 |
| Convenient | 278 | Folk house | 113 | Locality | 70 |
| Amuse oneself | 275 | Building | 110 | House | 63 |
| Rockery | 272 | Peace and happiness | 110 | It’s worth a go | 61 |
| Take a picture | 272 | Entrance ticket | 109 | Library building | 60 |
| Jixiao Villa | 264 | Come into | 82 | Visit | 57 |
| Explain | 242 | Be affected by | 82 | Hotel | 53 |
