# Supplementary material for: Post-occupancy evaluation of former residences of celebrities in Yangzhou based on the comparison of online reviews and questionnaire data
Source: PLoS One. 2026 Sep 3;21(9):e0357793. doi: 10.1371/journal.pone.0357793 (PMC13541147; doi:10.1371/journal.pone.0357793)
Supplement: Supplementary file 2 [file pone.0357793.s002.docx]

**AHP-FCE Evaluation Model, Data and Calculation Results**

for Post-occupancy Evaluation of Former Residences of Celebrities in Yangzhou

This study adopts a combined method of Analytic Hierarchy Process (AHP) and Fuzzy Comprehensive Evaluation (FCE) to conduct questionnaire surveys on former residences of celebrities in Yangzhou. AHP is used to determine the weight of each indicator, and FCE is used to handle the fuzziness of evaluation information and calculate the comprehensive score. The specific calculation steps and results are as follows:

**1 Indicator Weight Determination by AHP**

**（1）Establishment of Hierarchical Structure Model**

The evaluation objectives are divided into three levels: target layer, criterion layer, and sub- criterion layer (Table S1-1).

Table S1-1 Hierarchical Structure Model of Evaluation

| Target Layer | Criterion Layer | Sub- criterion Layer |
| --- | --- | --- |
| $A$ Evaluation of former residences of celebrities in Yangzhou | $A_{1}$ Attraction development | $a_{1}$ Attractiveness |
|  |  | $a_{2}$ Spatial planning |
|  |  | $a_{3}$ Function utilization |
|  |  | $a_{4}$ Exhibition content and form |
|  |  | $a_{5}$ Architecture preservation |
|  |  | $a_{6}$ Preservation of interior artifacts |
|  |  | $a_{7}$ External environmental development |
|  | $A_{2}$ Service and management | $a_{8}$ Consumption costs and payment methods |
|  |  | $a_{9}$ Consumer safety |
|  |  | $a_{10}$ Management practices |
|  |  | $a_{11}$ Staff attitude and professional level |
|  |  | $a_{12}$ Guide service |
|  |  | $a_{13}$ Online service |
|  | $A_{3}$ Environment and facilities | $a_{14}$ Infrastructure |
|  |  | $a_{15}$ Signage installation |
|  |  | $a_{16}$ Attraction accessibility |
|  |  | $a_{17}$ Parking facilities |
|  |  | $a_{18}$ Surrounding supporting facilities |
|  |  | $a_{19}$ Hygiene |
|  | $A_{4}$ Atmosphere and activities | $a_{20}$ Environmental atmosphere |
|  |  | $a_{21}$ Fun and interactivity |
|  |  | $a_{22}$ Activity diversity |
|  |  | $a_{23}$ Night tour programs |
|  |  | $a_{24}$ Development of unique advantages |
|  | $A_{5}$ Characteristics and exhibition of celebrity culture | $a_{25}$ Demonstration of cultural-educational value |
|  |  | $a_{26}$ Innovative expression methods |
|  |  | $a_{27}$ Characteristics of celebrities and exhibition forms |
|  |  | $a_{28}$ Promotion and recognition of celebrity culture |

**（2）Construction of Judgment Matrix**

Fifteen experts were invited to conduct pairwise comparisons of indicators at the same level using the 1–7 scale method (1=Equally Important, 3=Slightly Important, 5=Obviously Important, 7=Absolutely Important; 2, 4, 6 are intermediate values). For the criterion layer$A_{1},A_{2},...,A_{m}$，a judgment matrix$A=(a_{ij})_{m\times m}$is constructed, where $a_{ij}>0$，$a_{ji}=1/a_{ij}$，and $a_{ii}=1$。

**（3）Calculation of Weight Vector**

The geometric mean method (root method) is used to calculate weights:

Calculate the geometric mean of elements in each row of the judgment matrix:

$$\bar{w}_{i}=\left( \prod_{j=1}^{m} a_{ij} \right)^{1/m},i=1,\ldots,m$$

Normalize to obtain the weight vector:

$$w_{i}=\frac{\bar{w}_{i}}{\sum_{k=1}^{m} \bar{w}_{k}}$$

For the individual judgment matrices of 15 experts, the geometric mean of each comparison position $a_{ij}$ is first calculated to obtain the aggregated judgment matrix, and then the weights are calculated as above (Table S1-2).

In Table S1-2, the Weight column for the criterion layer denotes the global weight of that layer. The Local weights for the sub‑ criterion layer reflect the relative importance of each indicator within its own criterion group. The Global weights are then obtained by successive aggregation from the top level downwards via the formula $W_{\text{global}}=W_{\text{criteri}\text{on}}\times w_{\text{local}}$, and they indicate the ultimate impact weight of each sub‑indicator on the overall objective.

Table S1-2 Indicator Weight Results

| Target Layer | Weight | Criterion Layer | Weight | Sub- criterion Layer | Local weights | Global weights |
| --- | --- | --- | --- | --- | --- | --- |
| $A$ Evaluation of former residences of celebrities in Yangzhou | 1.0000 | $A_{1}$ Attraction development | 0.25201 | $a_{1}$ Attractiveness | 0.14325 | 0.03610 |
|  |  |  |  | $a_{2}$ Spatial planning | 0.14187 | 0.03575 |
|  |  |  |  | $a_{3}$ Function utilization | 0.13849 | 0.03490 |
|  |  |  |  | $a_{4}$ Exhibition content and form | 0.10278 | 0.02590 |
|  |  |  |  | $a_{5}$ Architecture preservation | 0.24127 | 0.06080 |
|  |  |  |  | $a_{6}$ Preservation of interior artifacts | 0.11171 | 0.02815 |
|  |  |  |  | $a_{7}$ External environmental development | 0.12063 | 0.03040 |
|  |  | $A_{2}$ Service and management | 0.16767 | $a_{8}$ Consumption costs and payment methods | 0.23890 | 0.04006 |
|  |  |  |  | $a_{9}$ Consumer safety | 0.09264 | 0.01553 |
|  |  |  |  | $a_{10}$ Management practices | 0.19811 | 0.03322 |
|  |  |  |  | $a_{11}$ Staff attitude and professional level | 0.16036 | 0.02689 |
|  |  |  |  | $a_{12}$ Guide service | 0.19811 | 0.03322 |
|  |  |  |  | $a_{13}$ Online service | 0.11188 | 0.01876 |
|  |  | $A_{3}$ Environment and facilities | 0.12729 | $a_{14}$ Infrastructure | 0.11483 | 0.01462 |
|  |  |  |  | $a_{15}$ Signage installation | 0.13551 | 0.01725 |
|  |  |  |  | $a_{16}$ Attraction accessibility | 0.31335 | 0.03989 |
|  |  |  |  | $a_{17}$ Parking facilities | 0.16748 | 0.02132 |
|  |  |  |  | $a_{18}$ Surrounding supporting facilities | 0.14444 | 0.01839 |
|  |  |  |  | $a_{19}$ Hygiene | 0.12440 | 0.01583 |
|  |  | $A_{4}$ Atmosphere and activities | 0.16767 | $a_{20}$ Environmental atmosphere | 0.18602 | 0.03119 |
|  |  |  |  | $a_{21}$ Fun and interactivity | 0.21602 | 0.03622 |
|  |  |  |  | $a_{22}$ Activity diversity | 0.24760 | 0.04152 |
|  |  |  |  | $a_{23}$ Night tour programs | 0.10275 | 0.01723 |
|  |  |  |  | $a_{24}$ Development of unique advantages | 0.24760 | 0.04152 |
|  |  | $A_{5}$ Characteristics and exhibition of celebrity culture | 0.28535 | $a_{25}$ Demonstration of cultural-educational value | 0.33333 | 0.09512 |
|  |  |  |  | $a_{26}$ Innovative expression methods | 0.16667 | 0.04756 |
|  |  |  |  | $a_{27}$ Characteristics of celebrities and exhibition forms | 0.33333 | 0.09512 |
|  |  |  |  | $a_{28}$ Promotion and recognition of celebrity culture | 0.16667 | 0.04756 |

Weights are computed from the judgment matrix below.

$A$ Evaluation of former residences of celebrities in Yangzhou AHP judgment matrix

| AHP judgment matrix | $A_{1}$ | $A_{2}$ | $A_{3}$ | $A_{4}$ | $A_{5}$ |
| --- | --- | --- | --- | --- | --- |
| $A_{1}$ | 1.00 | 2.00 | 2.00 | 1.00 | 1.00 |
| $A_{2}$ | 0.50 | 1.00 | 2.00 | 1.00 | 0.50 |
| $A_{3}$ | 0.50 | 0.50 | 1.00 | 1.00 | 0.50 |
| $A_{4}$ | 1.00 | 1.00 | 1.00 | 1.00 | 0.50 |
| $A_{5}$ | 1.00 | 2.00 | 2.00 | 2.00 | 1.00 |

$A_{1}$ Attraction development AHP judgment matrix

| AHP judgment matrix | $a_{1}$ | $a_{2}$ | $a_{3}$ | $a_{4}$ | $a_{5}$ | $a_{6}$ | $a_{7}$ |
| --- | --- | --- | --- | --- | --- | --- | --- |
| $a_{1}$ | 1.00 | 2.00 | 0.50 | 2.00 | 0.50 | 1.00 | 1.00 |
| $a_{2}$ | 0.50 | 1.00 | 1.00 | 2.00 | 0.50 | 2.00 | 1.00 |
| $a_{3}$ | 2.00 | 1.00 | 1.00 | 1.00 | 0.50 | 1.00 | 1.00 |
| $a_{4}$ | 0.50 | 0.50 | 1.00 | 1.00 | 0.50 | 1.00 | 1.00 |
| $a_{5}$ | 2.00 | 2.00 | 2.00 | 2.00 | 1.00 | 2.00 | 2.00 |
| $a_{6}$ | 1.00 | 0.50 | 1.00 | 1.00 | 0.50 | 1.00 | 1.00 |
| $a_{7}$ | 1.00 | 1.00 | 1.00 | 1.00 | 0.50 | 1.00 | 1.00 |

$A_{2}$ Service and management AHP judgment matrix

| AHP judgment matrix | $a_{8}$ | $a_{9}$ | $a_{10}$ | $a_{11}$ | $a_{12}$ | $a_{13}$ |
| --- | --- | --- | --- | --- | --- | --- |
| $a_{8}$ | 1.00 | 3.00 | 1.00 | 2.00 | 1.00 | 2.00 |
| $a_{9}$ | 0.33333 | 1.00 | 0.50 | 0.50 | 0.50 | 1.00 |
| $a_{10}$ | 1.00 | 2.00 | 1.00 | 1.00 | 1.00 | 2.00 |
| $a_{11}$ | 0.50 | 2.00 | 1.00 | 1.00 | 1.00 | 1.00 |
| $a_{12}$ | 1.00 | 2.00 | 1.00 | 1.00 | 1.00 | 2.00 |
| $a_{13}$ | 0.50 | 1.00 | 0.50 | 1.00 | 0.50 | 1.00 |

$A_{3}$ Environment and facilities AHP judgment matrix

| AHP judgment matrix | $a_{14}$ | $a_{15}$ | $a_{16}$ | $a_{17}$ | $a_{18}$ | $a_{19}$ |
| --- | --- | --- | --- | --- | --- | --- |
| $a_{14}$ | 1.00 | 1.00 | 0.33333 | 0.50 | 1.00 | 1.00 |
| $a_{15}$ | 1.00 | 1.00 | 0.50 | 1.00 | 1.00 | 1.00 |
| $a_{16}$ | 3.00 | 2.00 | 1.00 | 2.00 | 3.00 | 2.00 |
| $a_{17}$ | 2.00 | 1.00 | 0.50 | 1.00 | 2.00 | 1.00 |
| $a_{18}$ | 1.00 | 1.00 | 0.33333 | 0.50 | 1.00 | 2.00 |
| $a_{19}$ | 1.00 | 1.00 | 0.50 | 1.00 | 0.50 | 1.00 |

$A_{4}$ Atmosphere and activities AHP judgment matrix

| AHP judgment matrix | $a_{20}$ | $a_{21}$ | $a_{22}$ | $a_{23}$ | $a_{24}$ |
| --- | --- | --- | --- | --- | --- |
| $a_{20}$ | 1.00 | 1.00 | 0.50 | 3.00 | 0.50 |
| $a_{21}$ | 1.00 | 1.00 | 1.00 | 2.00 | 1.00 |
| $a_{22}$ | 2.00 | 1.00 | 1.00 | 2.00 | 1.00 |
| $a_{23}$ | 0.333333 | 0.50 | 0.50 | 1.00 | 0.50 |
| $a_{24}$ | 2.00 | 1.00 | 1.00 | 2.00 | 1.00 |

$A_{5}$ Characteristics and exhibition of celebrity culture AHP judgment matrix

| AHP judgment matrix | $a_{25}$ | $a_{26}$ | $a_{27}$ | $a_{28}$ |
| --- | --- | --- | --- | --- |
| $a_{25}$ | 1.00 | 2.00 | 1.00 | 2.00 |
| $a_{26}$ | 0.50 | 1.00 | 0.50 | 1.00 |
| $a_{27}$ | 1.00 | 2.00 | 1.00 | 2.00 |
| $a_{28}$ | 0.50 | 1.00 | 0.50 | 1.00 |

**（4）Consistency Test**

Calculate the maximum eigenvalue $\lambda_{\max}$ of the judgment matrix:

$$\lambda_{\max}=\frac{1}{m}\sum_{i=1}^{m} \frac{\left( \mathrm{Aw})_{i} \right.}{w_{i}}$$

Where $w=(w_{1},w_{2},...,w_{m})^{T}$，and $\left( \mathrm{Aw})_{i} \right.$ is the $i$-th component of the vector $Aw.$

Calculate the Consistency Index:

$$CI=\frac{\lambda_{\max}-m}{m-1}$$

Calculate the Consistency Ratio with the Random Consistency Index (Table S1-3):

| $CR=\frac{CI}{RI}$Table S1-3 Random Consistency Index (RI) | | | | | | | | | | | | | | |
| --- | --- | --- | --- | --- | --- | --- | --- | --- | --- | --- | --- | --- | --- | --- |
| *Order m* | 3 | 4 | 5 | 6 | 7 | 8 | 9 | 10 | 11 | 12 | 13 | 14 | 15 | 16 |
| *RI* | 0.52 | 0.89 | 1.12 | 1.26 | 1.36 | 1.41 | 1.46 | 1.49 | 1.52 | 1.54 | 1.56 | 1.58 | 1.59 | 1.5943 |
| *Order m* | 17 | 18 | 19 | 20 | 21 | 22 | 23 | 24 | 25 | 26 | 27 | 28 | 29 | 30 |
| *RI* | 1.6064 | 1.6133 | 1.6207 | 1.6292 | 1.6358 | 1.6403 | 1.6462 | 1.6497 | 1.6556 | 1.6587 | 1.6631 | 1.6670 | 1.6693 | 1.6724 |

Taking the judgment matrix of the criterion layer($A_{1}$-$A_{5}$) as an example, the maximum eigenvalue is calculated as $\lambda_{max}=5.117$.According to the formula $CI=\frac{\lambda_{max}-m}{m-1}$*，*substituting $m=5$，gives：

$$CI=\frac{5.117-5}{5-1}=\frac{0.117}{4}=0.02925$$

Referring to *Table S1-3*, for $m=5$, the random index is $RI=1.12$*.* Therefore:

$$CR=\frac{CI}{RI}=\frac{0.02925}{1.12}=0.0261<0.1$$

This satisfies the consistency requirement.

A judgment matrix is considered to have satisfactory consistency when $CR<0.1.$

All judgment matrices in this study pass the consistency test $CR<0.1$ (Table S1-4).

Table S1-4 Consistency Test Results of Judgment Matrices at All Levels

| Judgment Matrix | Order *m* | $\lambda_{\text{max}}$ | $CI$ | $RI$ | $CR$ | Pass ($CR<0.1$) |
| --- | --- | --- | --- | --- | --- | --- |
| Criterion Layer | 5 | 5.117 | 0.02923 | 1.12 | 0.0261 | Yes |
| Sub-layer of Attraction development | 7 | 7.277 | 0.04613 | 1.36 | 0.0339 | Yes |
| Sub-layer of Service and management | 6 | 6.083 | 0.01670 | 1.26 | 0.0133 | Yes |
| Sub-layer of Environment and facilities | 6 | 6.178 | 0.03560 | 1.26 | 0.0283 | Yes |
| Sub-layer of Atmosphere and activities | 5 | 5.146 | 0.03643 | 1.12 | 0.0325 | Yes |
| Sub-layer of Characteristics and exhibition of celebrity culture | 4 | 4.000 | 0.00000 | 0.89 | 0.0000 | Yes |

**2 Calculation of Fuzzy Comprehensive Evaluation (FCE)**

(1) **Determination of Comment Set and Membership Function**

The comment set $V=\{v_{1},v_{2},v_{3},v_{4},v_{5}\}$ orresponds to Very Satisfied, Satisfied, Neutral, Dissatisfied, Very Dissatisfied, with the score vector:

$$V=[100,80,60,40,20]^{T}$$

Based on 1,643 valid questionnaires, the frequency$n_{ijk}$（$k=1,...,5$）of each comment level is counted to obtain the membership degree:

$$r_{ijk}=\frac{n_{ijk}}{N}(N=1643)$$

The fuzzy evaluation vector of the sub-indicator：

$$R_{ij}=[r_{ij1},r_{ij2},r_{ij3},r_{ij4},r_{ij5}]$$

The membership vectors (frequencies) of 28 sub- criterion layer indicators are shown in Table S1-5.

Table S1-5 Membership Vectors (Fuzzy Evaluation Frequencies) of Each Indicator

| Indicator | Very Satisfied | Satisfied | Neutral | Dissatisfied | Very Dissatisfied |
| --- | --- | --- | --- | --- | --- |
| $a_{1}$ Attractiveness | 0.0018 | 0.4656 | 0.5313 | 0.0006 | 0.0006 |
| $a_{2}$ Spatial planning | 0.0030 | 0.4242 | 0.5703 | 0.0018 | 0.0006 |
| $a_{3}$ Function utilization | 0.0122 | 0.3116 | 0.6586 | 0.0116 | 0.0061 |
| $a_{4}$ Exhibition content and form | 0.0043 | 0.0061 | 0.9483 | 0.0341 | 0.0073 |
| $a_{5}$ Architecture preservation | 0.0645 | 0.8984 | 0.0128 | 0.0122 | 0.0122 |
| $a_{6}$ Preservation of interior artifacts | 0.0061 | 0.1217 | 0.8016 | 0.0627 | 0.0079 |
| $a_{7}$ External environmental development | 0.1339 | 0.6147 | 0.2343 | 0.0122 | 0.0049 |
| $a_{8}$ Consumption costs and payment methods | 0.0037 | 0.0858 | 0.8113 | 0.0858 | 0.0134 |
| $a_{9}$ Consumer safety | 0.2228 | 0.6586 | 0.0846 | 0.0243 | 0.0097 |
| $a_{10}$ Management practices | 0.0274 | 0.4772 | 0.4753 | 0.0152 | 0.0049 |
| $a_{11}$ Staff attitude and professional level | 0.0390 | 0.5076 | 0.4334 | 0.0164 | 0.0037 |
| $a_{12}$ Guide service | 0.0177 | 0.4060 | 0.5514 | 0.0195 | 0.0055 |
| $a_{13}$ Online service | 0.0183 | 0.3348 | 0.6135 | 0.0274 | 0.0061 |
| $a_{14}$ Infrastructure | 0.0542 | 0.4735 | 0.4498 | 0.0189 | 0.0037 |
| $a_{15}$ Signage installation | 0.0359 | 0.0116 | 0.4869 | 0.4017 | 0.0639 |
| $a_{16}$ Attraction accessibility | 0.0012 | 0.0809 | 0.4443 | 0.3749 | 0.0986 |
| $a_{17}$ Parking facilities | 0.0049 | 0.0590 | 0.5697 | 0.3293 | 0.0371 |
| $a_{18}$ Surrounding supporting facilities | 0.0079 | 0.0584 | 0.5167 | 0.3731 | 0.0438 |
| $a_{19}$ Hygiene | 0.0913 | 0.6415 | 0.2240 | 0.0402 | 0.0030 |
| $a_{20}$ Environmental atmosphere | 0.0134 | 0.1412 | 0.7961 | 0.0426 | 0.0067 |
| $a_{21}$ Fun and interactivity | 0.0067 | 0.0682 | 0.4029 | 0.4614 | 0.0609 |
| $a_{22}$ Activity diversity | 0.0189 | 0.0475 | 0.3457 | 0.5307 | 0.0572 |
| $a_{23}$ Night tour programs | 0.0146 | 0.0736 | 0.7401 | 0.1400 | 0.0316 |
| $a_{24}$ Development of unique advantages | 0.0158 | 0.0353 | 0.4851 | 0.4114 | 0.0523 |
| $a_{25}$ Demonstration of cultural-educational value | 0.0024 | 0.0572 | 0.7657 | 0.1436 | 0.0310 |
| $a_{26}$ Innovative expression methods | 0.0079 | 0.0353 | 0.4820 | 0.4218 | 0.0530 |
| $a_{27}$ Characteristics of celebrities and exhibition forms | 0.0073 | 0.0262 | 0.8831 | 0.0743 | 0.0091 |
| $a_{28}$ Promotion and recognition of celebrity culture | 0.0097 | 0.0481 | 0.8977 | 0.0359 | 0.0085 |

**（2）First-level Fuzzy Comprehensive Evaluation (Criterion Layer Evaluation)**

Let the $i$-th criterion layer have $m_{i}$ sub-indicators, with the weight vector $\mathbf{w}_{i}=[w_{i1},w_{i2},...,w_{im_{i}}]$) ($\sum_{j=1}^{m_{i}} w_{ij}=1)$. The fuzzy evaluation vectors of each sub-indicator are combined into a matrix:

$$\boldsymbol{R}_{i}=\left[ \begin{aligned} R_{i1} \\ R_{i2} \\ \vdots\\ R_{im_{i}} \end{aligned} \right]\in\mathbb{R}^{m_{i}\times5}$$

The weighted average fuzzy operator $M(\cdot,\oplus)$ (ordinary matrix multiplication) is used to calculate the fuzzy comprehensive evaluation vector of the criterion layer:

$$\boldsymbol{B}_{i}=\boldsymbol{w}_{i}\circ\boldsymbol{R}_{i}=[b_{i1},b_{i2},...,b_{i5}]$$

Where：

$$b_{ik}=\sum_{j=1}^{m_{i}} w_{ij}\cdot r_{ijk},k=1,...,5$$

The quantitative score of the criterion layer is:：

$$F_{i}=\boldsymbol{B}_{i}\cdot V=\sum_{k=1}^{5} b_{ik}\cdot V_{k}$$

**Criterion Layer *A₁* (Attraction development)**
The weight of this criterion is $W_{1}=0.25201$. It includes indicators $a_{1}$ through $a_{7}$, with the local weight vector:

$$W_{1}=(0.14325, 0.14187, 0.13849, 0.10278, 0.24127, 0.11171, 0.12063)$$

Substituting the membership degrees of $a_{1}$ to $a_{7}$ into the fuzzy relation matrix $R_{1}$, we obtain the synthetic evaluation vector $B_{1}=W_{1}\times R_{1}$:

| Grade | Very Satisfied | Satisfied | Neutral | Dissatisfied | Very Dissatisfied |
| --- | --- | --- | --- | --- | --- |
| $B_{1}$ | 0.0352 | 0.4752 | 0.4666 | 0.0169 | 0.0062 |

The percentage score for criterion $A_{1}$ is:

$$\begin{matrix} F_{A₁} & =0.0352\times100+0.4752\times80+0.4666\times60+0.0169\times40+0.0062\times20 \\ & =\boldsymbol{70.33} \\ & \end{matrix}$$

**Criterion Layer *A₂* (Service and management)**
The weight of this criterion is $W_{2}=0.16767$*.* It includes indicators $a_{8}$ through $a_{13}$, with the local weight vector:

$$W_{2}=(0.23890, 0.09264, 0.19811, 0.16036, 0.19811, 0.11188)$$

Synthetic evaluation vector $B_{2}=W_{2}\times R_{2}$*:*

| Grade | Very Satisfied | Satisfied | Neutral | Dissatisfied | Very Dissatisfied |
| --- | --- | --- | --- | --- | --- |
| $B_{2}$ | 0.0388 | 0.3753 | 0.5432 | 0.0353 | 0.0074 |

Percentage score for $A_{2}$*:*

$$\begin{matrix} F_{A2} & =0.0388\times100+0.3753\times80+0.5432\times60+0.0353\times40+0.0074\times20 \\ & =\boldsymbol{68.05} \\ & \end{matrix}$$

**Criterion Layer *A₃* (Environment and facilities)**
The weight of this criterion is $W_{3}=0.12729$*.* It includes indicators $a_{14}$ through $a_{19}$, with the local weight vector:

$$W_{3}=(0.11483, 0.13551, 0.31335, 0.16748, 0.14444, 0.12440)$$

Synthetic evaluation vector $B_{3}=W_{3}\times R_{3}$*:*

| Grade | Very Satisfied | Satisfied | Neutral | Dissatisfied | Very Dissatisfied |
| --- | --- | --- | --- | --- | --- |
| $B_{3}$ | 0.0248 | 0.1794 | 0.4548 | 0.2881 | 0.0529 |

Percentage score for $A_{3}$*:*

$$\begin{matrix} F_{A3} & =0.0248\times100+0.1794\times80+0.4548\times60+0.2881\times40+0.0529\times20 \\ & =\boldsymbol{56.70} \\ & \end{matrix}$$

**Criterion Layer *A₄* (Atmosphere and activities)**
The weight of this criterion is $W_{4}=0.16767$*.* It includes indicators $a_{20}$ through $a_{24}$, with the local weight vector:

$$W_{4}=(0.18602, 0.21602, 0.24760, 0.10275, 0.24760)$$

Synthetic evaluation vector $B_{4}=W_{4}\times R_{4}$*:*

| Grade | Very Satisfied | Satisfied | Neutral | Dissatisfied | Very Dissatisfied |
| --- | --- | --- | --- | --- | --- |
| $B_{4}$ | 0.0140 | 0.0691 | 0.5169 | 0.3552 | 0.0448 |

Percentage score for $A_{4}$*:*

$$\begin{matrix} F_{A4} & =0.0140\times100+0.0691\times80+0.5169\times60+0.3552\times40+0.0448\times20 \\ & =\boldsymbol{53.05} \\ & \end{matrix}$$

**Criterion Layer *A₅* (Characteristics and exhibition of celebrity culture)**
The weight of this criterion is $W_{5}=0.28535$*.* It includes indicators $a_{25}$ through $a_{28}$, with the local weight vector:

$$W_{5}=(0.33333, 0.16667, 0.33333, 0.16667)$$

Synthetic evaluation vector $B_{5}=W_{5}\times R_{5}$*:*

| Grade | Very Satisfied | Satisfied | Neutral | Dissatisfied | Very Dissatisfied |
| --- | --- | --- | --- | --- | --- |
| $B_{5}$ | 0.0062 | 0.0417 | 0.7796 | 0.1489 | 0.0236 |

Percentage score for $A_{5}$*:*

$$\begin{matrix} F_{A5} & =0.0062\times100+0.0417\times80+0.7796\times60+0.1489\times40+0.0236\times20 \\ & =\boldsymbol{57.16} \\ & \end{matrix}$$

**（3）Second-level Fuzzy Comprehensive Evaluation (Target Layer Evaluation)**

The five criterion‑layer evaluation vectors $B_{1}$ to $B_{5}$ form the fuzzy relation matrix $R_{Total}$ for the overall target:

$$R_{Total}=\left[ \begin{aligned} B_{1} \\ B_{2} \\ B_{3} \\ B_{4} \\ B_{5} \end{aligned} \right]=\left[ \begin{matrix} 0.0352 & 0.4752 & 0.4666 & 0.0169 & 0.0062 \\ 0.0388 & 0.3753 & 0.5432 & 0.0353 & 0.0074 \\ 0.0248 & 0.1794 & 0.4548 & 0.2881 & 0.0529 \\ 0.0140 & 0.0691 & 0.5169 & 0.3552 & 0.0448 \\ 0.0062 & 0.0417 & 0.7796 & 0.1489 & 0.0236 \end{matrix} \right]$$

The weight vector for the criterion layer is:

$$W_{Total}=(0.25201, 0.16767, 0.12729, 0.16767, 0.28535)$$

The overall fuzzy evaluation vector $B_{Total}=W_{Total}\times R_{Total}$ is computed as:

$$\begin{matrix} B_{Total} & =(0.25201,0.16767,0.12729,0.16767,0.28535)\times R_{Total} \\ & =(0.0226, 0.2290, 0.5757, 0.1489, 0.0238) \end{matrix}$$

The final overall percentage score is:

$$\begin{matrix} F_{Total} & =0.0226\times100+0.2290\times80+0.5757\times60+0.1489\times40+0.0238\times20 \\ & =\mathbf{61.56} \\ & \end{matrix}$$

The final computed results are presented in the following table：

Table S1-6 The final results

| **Target layer** | **Score** | **Criterion layer** | **Score** | **Sub-criterion layer** | **Score** |
| --- | --- | --- | --- | --- | --- |
| *A* Evaluation of former residences of celebrities in Yangzhou | 61.56 | $A_{1}$ Attraction development | 70.33 | $a_{1}$ Attractiveness | 69.35 |
|  |  |  |  | $a_{2}$ Spatial planning | 68.54 |
|  |  |  |  | $a_{3}$ Function utilization | 66.24 |
|  |  |  |  | $a_{4}$ Exhibition content and form | 59.32 |
|  |  |  |  | $a_{5}$ Architecture preservation | 79.82 |
|  |  |  |  | $a_{6}$ Preservation of interior artifacts | 61.11 |
|  |  |  |  | $a_{7}$ External environmental development | 77.21 |
|  |  | $A_{2}$ Service and management | 68.05 | $a_{8}$ Consumption costs and payment methods | 59.61 |
|  |  |  |  | $a_{9}$ Consumer safety | 81.21 |
|  |  |  |  | $a_{10}$ Management practices | 70.14 |
|  |  |  |  | $a_{11}$ Staff attitude and professional level | 71.23 |
|  |  |  |  | $a_{12}$ Guide service | 68.22 |
|  |  |  |  | $a_{13}$ Online service | 66.63 |
|  |  | $A_{3}$ Environment and facilities | 56.70 | $a_{14}$ Infrastructure | 71.11 |
|  |  |  |  | $a_{15}$ Signage installation | 51.08 |
|  |  |  |  | $a_{16}$ Attraction accessibility | 50.22 |
|  |  |  |  | $a_{17}$ Parking facilities | 53.31 |
|  |  |  |  | $a_{18}$ Surrounding supporting facilities | 52.27 |
|  |  |  |  | $a_{19}$ Hygiene | 75.55 |
|  |  | $A_{4}$ Atmosphere and activities | 53.05 | $a_{20}$ Environmental atmosphere | 62.24 |
|  |  |  |  | $a_{21}$ Fun and interactivity | 49.97 |
|  |  |  |  | $a_{22}$ Activity diversity | 48.80 |
|  |  |  |  | $a_{23}$ Night tour programs | 57.99 |
|  |  |  |  | $a_{24}$ Development of unique advantages | 51.02 |
|  |  | $A_{5}$ Characteristics and exhibition of celebrity culture | 57.16 | $a_{25}$ Demonstration of cultural-educational value | 57.13 |
|  |  |  |  | $a_{26}$ Innovative expression methods | 50.47 |
|  |  |  |  | $a_{27}$ Characteristics of celebrities and exhibition forms | 58.97 |
|  |  |  |  | $a_{28}$ Promotion and recognition of celebrity culture | 60.29 |
