# Supplementary material for: Post-occupancy evaluation of former residences of celebrities in Yangzhou based on the comparison of online reviews and questionnaire data
Source: PLoS One. 2026 Sep 3;21(9):e0357793. doi: 10.1371/journal.pone.0357793 (PMC13541147; doi:10.1371/journal.pone.0357793)
Supplement: Supplementary file 3 [file pone.0357793.s003.docx]

**Post-occupancy Evaluation (POE) Questionnaire for Former Residences of Celebrities in Yangzhou**

Dear Visitors,

We are conducting a visitor satisfaction survey on former residences of celebrities in Yangzhou (including Heyuan Garden, Geyuan Garden, Wang’s Residence, Lu’s Salt Merchant Residence, Zhu Ziqing’s Residence, Wu Daotai’s Residence and 9 other sites). Your genuine feedback will help improve the service quality and cultural experience of these heritage sites. This questionnaire is anonymous and used solely for academic research. Thank you for your cooperation!

**I. Basic Information**

1. Gender: □ Male □ Female
2. Age: □ Under 18 □ 18-30 □ 31-45 □ 46-60 □ Over 60
3. Education Level: □ Junior High School or Below □ Senior High School/Technical Secondary School □ Junior College/Undergraduate □ Master’s Degree or Above
4. Name of the Residence(s) Visited (Multiple Choice):
5. □ Heyuan Garden □ Geyuan Garden □ Wang’s Small Courtyard □ Lu’s Salt Merchant Residence
6. □ Zhu Ziqing’s Former Residence □ Wu Daotai’s Residence □ Xiaopangu □ Jia’s Salt Merchant Residence □ Yipu Garden
7. Visit Frequency: □ First Visit □ Second or More Visits

**II. Evaluation Section**

Please tick "√" under the corresponding option based on your actual experience.

Scoring Criteria: 5-Very Satisfied, 4-Satisfied, 3-Neutral, 2-Dissatisfied, 1-Very Dissatisfied

**1. Attraction development**

| Evaluation Indicators | 5 | 4 | 3 | 2 | 1 |
| --- | --- | --- | --- | --- | --- |
| 1. Attractiveness (landscape aesthetics, uniqueness, etc.) |  |  |  |  |  |
| 2. Spatial planning (rationality of spatial layout, etc.) |  |  |  |  |  |
| 3. Function utilization (rationality of usage, etc.) |  |  |  |  |  |
| 4. Exhibition content and form (rationality of interpretation boards, display methods, etc.) |  |  |  |  |  |
| 5. Architecture preservation (authenticity, integrity, etc.) |  |  |  |  |  |
| 6. Preservation of interior artifacts (completeness of cultural relics, etc.) |  |  |  |  |  |
| 7. External environmental development (greening, roads, surrounding style, etc.) |  |  |  |  |  |

**2. Service and management**

| Evaluation Indicators | 5 | 4 | 3 | 2 | 1 |
| --- | --- | --- | --- | --- | --- |
| 8. Consumption costs and payment methods (ticket price, payment convenience, etc.) |  |  |  |  |  |
| 9. Consumer safety (forced selling, arbitrary price changes, etc.) |  |  |  |  |  |
| 10. Management practices (opening hours, order maintenance, etc.) |  |  |  |  |  |
| 11. Staff attitude and professional level (service attitude, efficiency, etc.) |  |  |  |  |  |
| 12. Guide service (tour guides, audio guides, etc.) |  |  |  |  |  |
| 13. Online service (online reservation, information inquiry, etc.) |  |  |  |  |  |

**3. Environment and facilities**

| Evaluation Indicators | 5 | 4 | 3 | 2 | 1 |
| --- | --- | --- | --- | --- | --- |
| 14. Infrastructure (rest seats, trash cans, etc.) |  |  |  |  |  |
| 15. Signage installation (direction signs, maps, etc.) |  |  |  |  |  |
| 16. Attraction accessibility (transportation convenience, ease of finding, etc.) |  |  |  |  |  |
| 17. Parking facilities (parking convenience, number of parking spaces, etc.) |  |  |  |  |  |
| 18. Surrounding supporting facilities (catering, shops, rest areas, etc.) |  |  |  |  |  |
| 19. Hygiene (environmental cleanliness, cleaning quality, etc.) |  |  |  |  |  |

**4. Atmosphere and activities**

| Evaluation Indicators | 5 | 4 | 3 | 2 | 1 |
| --- | --- | --- | --- | --- | --- |
| 20. Environmental atmosphere (historical sense, cultural atmosphere, etc.) |  |  |  |  |  |
| 21. Fun and interactivity (attractiveness of experience programs, etc.) |  |  |  |  |  |
| 22. Activity diversity (variety of optional activities, etc.) |  |  |  |  |  |
| 23. Night tour programs (night opening, lighting, performances, etc.) |  |  |  |  |  |
| 24. Development of unique advantages (unique selling points, differentiation, etc.) |  |  |  |  |  |

**5. Characteristics and exhibition of celebrity culture**

| Evaluation Indicators | 5 | 4 | 3 | 2 | 1 |
| --- | --- | --- | --- | --- | --- |
| 25. Demonstration of cultural-educational value (knowledge acquisition, etc.) |  |  |  |  |  |
| 26. Innovative expression methods (multimedia, VR/AR, cultural and creative design, etc.) |  |  |  |  |  |
| 27. Characteristics of celebrities and exhibition forms (highlighting celebrity IP, etc.) |  |  |  |  |  |
| 28. Promotion and recognition of celebrity culture (publicity effect, etc.) |  |  |  |  |  |

**Expert Questionnaire on Indicator Weight of Post-occupancy Evaluation for Former Residences of Celebrities in Yangzhou**

Dear Experts,

We are conducting an Analytic Hierarchy Process (AHP) study on the indicator weight of post-occupancy evaluation for former residences of celebrities in Yangzhou. Please judge the relative importance of indicators at each level through pairwise comparisons based on your professional experience. Your judgment will directly affect the scientificity of indicator weights. We will strictly abide by academic ethics, use your responses only for academic research, and never disclose them for other purposes. Thank you very much for your participation!

**Expert Background**

Professional Field: __________

Years of Practice: ____ years

Professional Title/Position: __________

**Part I: Criteria Level Indicators (5 Dimensions in Total)**

Instructions

AHP Scoring Rules: Score pairwise comparisons of two different variables at the same level on a scale of 1–7.

Value Explanation: 1=Equally Important, 3=Slightly Important, 5=Obviously Important, 7=Absolutely Important; 2, 4, 6 are intermediate values. If the importance ratio of indicator *i* to indicator *j* is *A_ij_*, the reciprocal represents the importance of indicator *j* to indicator *i* (*A_ji_=1/A_ij_*).

**Comparison Matrix**

|  | $A_{1}$ Attraction development | $A_{2}$ Service and management | $A_{3}$ Environment and facilities | $A_{4}$ Atmosphere and activities | $A_{5}$ Characteristics and exhibition of celebrity culture |
| --- | --- | --- | --- | --- | --- |
| $A_{1}$ Attraction development |  |  |  |  |  |
| $A_{2}$ Service and management |  |  |  |  |  |
| $A_{3}$ Environment and facilities |  |  |  |  |  |
| $A_{4}$ Atmosphere and activities |  |  |  |  |  |
| $A_{5}$ Characteristics and exhibition of celebrity culture |  |  |  |  |  |

**Part II: Sub-criteria Level Indicators**

**(1) Attraction development**

|  | $a_{1}$ Attractiveness | $a_{2}$ Spatial planning | $a_{3}$ Function utilization | $a_{4}$ Exhibition content and form | $a_{5}$ Architecture preservation | $a_{6}$ Preservation of interior artifacts | $a_{7}$ External environmental development |
| --- | --- | --- | --- | --- | --- | --- | --- |
| $a_{1}$ Attractiveness |  |  |  |  |  |  |  |
| $a_{2}$ Spatial planning |  |  |  |  |  |  |  |
| $a_{3}$ Function utilization |  |  |  |  |  |  |  |
| $a_{4}$ Exhibition content and form |  |  |  |  |  |  |  |
| $a_{5}$ Architecture preservation |  |  |  |  |  |  |  |
| $a_{6}$ Preservation of interior artifacts |  |  |  |  |  |  |  |
| $a_{7}$ External environmental development |  |  |  |  |  |  |  |

**（2）Service and management**

|  | $a_{8}$ Consumption costs and payment methods | $a_{9}$ Consumer safety | $a_{10}$ Management practices | $a_{11}$ Staff attitude and professional level | $a_{12}$ Guide service | $a_{13}$ Online service |
| --- | --- | --- | --- | --- | --- | --- |
| $a_{8}$ Consumption costs and payment methods |  |  |  |  |  |  |
| $a_{9}$ Consumer safety |  |  |  |  |  |  |
| $a_{10}$ Management practices |  |  |  |  |  |  |
| $a_{11}$ Staff attitude and professional level |  |  |  |  |  |  |
| $a_{12}$ Guide service |  |  |  |  |  |  |
| $a_{13}$ Online service |  |  |  |  |  |  |

**（3）Environment and facilities**

|  | $a_{14}$ Infrastructure | $a_{15}$ Signage installation | $a_{16}$ Attraction accessibility | $a_{17}$ Parking facilities | $a_{18}$ Surrounding supporting facilities | $a_{19}$ Hygiene |
| --- | --- | --- | --- | --- | --- | --- |
| $a_{14}$ Infrastructure |  |  |  |  |  |  |
| $a_{15}$ Signage installation |  |  |  |  |  |  |
| $a_{16}$ Attraction accessibility |  |  |  |  |  |  |
| $a_{17}$ Parking facilities |  |  |  |  |  |  |
| $a_{18}$ Surrounding supporting facilities |  |  |  |  |  |  |
| $a_{19}$ Hygiene |  |  |  |  |  |  |

**（4）Atmosphere and activities**

|  | $a_{20}$ Environmental atmosphere | $a_{21}$ Fun and interactivity | $a_{22}$ Activity diversity | $a_{23}$ Night tour programs | $a_{24}$ Development of unique advantages |
| --- | --- | --- | --- | --- | --- |
| $a_{20}$ Environmental atmosphere |  |  |  |  |  |
| $a_{21}$ Fun and interactivity |  |  |  |  |  |
| $a_{22}$ Activity diversity |  |  |  |  |  |
| $a_{23}$ Night tour programs |  |  |  |  |  |
| $a_{24}$ Development of unique advantages |  |  |  |  |  |

**（5）Characteristics and exhibition of celebrity culture**

|  | $a_{25}$ Demonstration of cultural-educational value | $a_{26}$ Innovative expression methods | $a_{27}$ Characteristics of celebrities and exhibition forms | $a_{28}$ Promotion and recognition of celebrity culture |
| --- | --- | --- | --- | --- |
| $a_{25}$ Demonstration of cultural-educational value |  |  |  |  |
| $a_{26}$ Innovative expression methods |  |  |  |  |
| $a_{27}$ Characteristics of celebrities and exhibition forms |  |  |  |  |
| $a_{28}$ Promotion and recognition of celebrity culture |  |  |  |  |
