# Supplementary material for: Post-occupancy evaluation of former residences of celebrities in Yangzhou based on the comparison of online reviews and questionnaire data
Source: PLoS One. 2026 Sep 3;21(9):e0357793. doi: 10.1371/journal.pone.0357793 (PMC13541147; doi:10.1371/journal.pone.0357793)
Supplement: Supplementary file 4 [file pone.0357793.s004.docx]

**Comparison of occurrence frequencies of online review categories between winter and non‑winter**

Online reviews were divided into two subsets: winter (December-February) and non-winter (March-November). The occurrence frequencies of the five core negative categories were separately calculated, and a chi‑square test was performed to compare the distribution between the two groups (Table 1).

**Table S3-1 Chi‑square test results for seasonal differences**

| **Core category** | **Winter frequency** | **Nonwinter frequency** | **Total** | **Expected winter frequency** | **Expected nonwinter frequency** |
| --- | --- | --- | --- | --- | --- |
| **Accessibility and exhibition design** | 398 | 843 | 1,241 | 394.96 | 846.04 |
| **Management, operation and maintenance** | 172 | 377 | 549 | 174.73 | 374.27 |
| **Facility development** | 139 | 305 | 444 | 141.31 | 302.69 |
| **Tourist experience and activity engagement** | 346 | 733 | 1,079 | 343.40 | 735.60 |
| **Celebrity culture exploration and representation** | 205 | 441 | 646 | 205.60 | 440.40 |
| **Total** | 1,260 | 2,699 | 3,959 | 1,260.00 | 2,699.00 |

Expected frequencies are calculated using the chi‑square formula $E_{ij}=\frac{R_{i}\times C_{j}}{N}$*,* where $R_{i}$ is the row total, $C_{j}$ is the column total, and $N$ is the grand total. The degrees of freedom for this test are $df=(r-1)(c-1)=(2-1)(5-1)=4$*.*

Note: All expected frequencies are rounded to two decimal places.

The results show that χ²(4) ≈ 0.19, p ≈ 0.996, indicating no significant difference in the distribution of core negative themes between winter and non‑winter, suggesting that seasonal factors exert limited structural influence on the core evaluation dimensions.
